# Risky Drinking in Midlife Men: Insights From Australia's National Drug Strategy Household Survey

**DOI:** 10.1111/dar.70149

**Published:** 2026-04-13

**Authors:** Stefano Zaccagnini, Ashlea Bartram, Michael Livingston, James A. Smith, Nataly Bovopoulos, Jacqueline Bowden

**Affiliations:** ^1^ National Centre for Education and Training on Addiction Flinders University Adelaide Australia; ^2^ College of Medicine and Public Health, Flinders Health and Medical Research Institute Flinders University Adelaide Australia; ^3^ National Drug Research Institute Curtin University Perth Australia; ^4^ Centre for Alcohol Policy Research La Trobe University Melbourne Australia; ^5^ Rural and Remote Health Flinders University Darwin Australia; ^6^ Alcohol and Drug Foundation Melbourne Australia

**Keywords:** alcohol, Australia, factors, midlife men, national guidelines, survey

## Abstract

**Introduction:**

Australian midlife men are more likely to drink exceeding Australia's Alcohol guidelines for adults compared to midlife women. Despite this, there is a paucity of research investigating the factors associated with risky drinking for midlife men to inform preventive health efforts. This study investigates these factors and how they differ between younger and older midlife men.

**Methods:**

Secondary data analysis was conducted using the 2019 and 2022/23 waves of the National Drug Strategy Household Survey. Data for midlife men (aged 30–59 years, *n* = 6471) were analysed, comprising younger (aged 30–44 years, *n* = 3311) and older (45–59 years, *n* = 3160) midlife age groups. Associations between behavioural, psychological and demographic factors and risky drinking, and interactions with midlife age groups were examined using chi‐square and multivariable logistic regression analyses.

**Results:**

Overall, 42.9% of midlife men reported risky drinking. Past year tobacco (OR = 1.70, CI = 1.35–2.14) and illicit/non‐medicinal drug use (OR = 3.54, CI = 2.92–4.31), residing in Rural/Remote/Regional locations (OR = 1.76, CI = 1.48–2.11), higher psychological distress scores (OR = 1.37, CI = 1.05–1.79), working managerial (OR = 1.48, CI = 1.14–1.91) or tech/trade (OR = 1.48, CI = 1.17–1.87) occupations, and higher household earnings (OR = 1.70, CI = 1.41–2.06) were all significantly associated with risky drinking. Greater psychological distress scores were significantly associated with risky drinking among younger (OR = 0.49, CI = 0.32–0.75), but not older midlife men, while being married/de facto relationship was significantly associated with risky drinking among older (OR = 1.55, CI = 1.01–2.39), but not younger midlife men.

**Discussion and Conclusion:**

This study highlights a range of modifiable and non‐modifiable factors that may inform the development of future preventive health policy and practise strategies for this high prevalence group.

## 1 Introduction

Globally, alcohol is a primary contributor to negative health and wellbeing outcomes, linked to over 200 different injuries, harms and diseases [1] and can result in the development of both short‐ and long‐term health concerns [2, 3, 4, 5]. Alcohol consumption has become normative and widespread in many societies and cultures [5, 6], with men often identified as being more likely to engage in risky alcohol consumption [7]. The risky consumption of alcohol is particularly problematic among group settings dominated by men [8, 9, 10, 11], often conforming to health‐harming masculine norms or traits associated with hegemonic masculinity [8, 12].

Alcohol‐related harms have contributed to approximately 2.6 million deaths among men and women worldwide in 2019, with 77% of deaths occurring in men [13]. In recent decades, rates of risky drinking among men in most high‐income countries have continued to decline [7, 14]. For Australia specifically, recent data from the National Drug Strategy Household Survey (NDSHS) [15] has shown that approximately 31% of individuals aged 14 years and above have consumed alcohol above recommended levels based on current national guidelines [16]. This rate has declined since 2007, where 38% of Australian men have reported drinking that exceeded recommended levels [16]. Despite this continued declining trend, the risks and harms associated with alcohol consumption continue to remain higher among men [1, 7, 17]. Primary harms of risky drinking to self and others include, but are not limited to, traffic accidents, domestic and family violence, poorer mental health and wellbeing [18, 19, 20, 21], and adverse physical health conditions including cardiovascular diseases and cancers [18, 19, 22]. The Australian Institute of Health and Welfare identified alcohol as the fourth highest risk factor contributing to the burden of disease for men overall, with 5.5% of Disability‐Adjusted Life Years attributed to alcohol consumption [23]. As such, alcohol contributed to approximately 1182 deaths for Australian men in 2023, with mortality rates higher among older men [23].

Australian studies exploring alcohol trends have previously found that consumption among men typically peaks in midlife, often involving frequent and routinised drinking habits as opposed to occasional binge drinking [16, 24, 25]. This generally remains high until older adulthood where drinking often declines [25]. A recent Australian study conducted by Leggat and colleagues analysed Australian longitudinal survey data, demonstrating that frequent drinking trajectories peak among midlife men aged between 45 and 64 years [25]. Similarly, the Ten to Men Australian longitudinal study further highlights that men aged 45–57 years at baseline had also engaged in frequent drinking the most, with consumption trends presenting minimal shifts over time within this age group [24].

Studies investigating risky drinking and prevention in Australia have focused predominantly on younger populations [26, 27, 28, 29], with a growing body of research beginning to examine older populations [30, 31, 32, 33]. However, Australian studies explicitly investigating midlife populations have predominantly focused on women through qualitative approaches [34, 35, 36, 37, 38]. In contrast, studies focussing on investigating alcohol use among midlife men currently remain scarce, with most existing single‐gender studies for men being either conducted outside of Australia [12, 39, 40] or focused on the health outcomes for men who drink and other factors that categorise within the medical discipline [34]. A recent review and editorial have examined the foci for existing single‐gender alcohol studies and highlighted there was an important need for research focusing on alcohol consumption and determinants among midlife men [34, 41]. While some international mixed‐gender studies include midlife men within their sampling, these studies employ qualitative designs to prioritise other themes and objectives, including reasons to justify routine drinking [42], home drinking practises and perceptions [30, 43], drinking and loneliness [44] and a review investigating the motivations and constructions of non‐problematised alcohol consumption among midlife populations [45]. For single‐gendered investigations on midlife men internationally, studies have typically been of qualitative focus, including a review exploring meanings and experiences behind alcohol engagement [40] and the understandings of health harms accompanying alcohol consumption [39]. Despite this, investigations and overviews into specific factors linked with risky drinking and midlife men through a quantitative analysis are few, both internationally and within Australian settings.

Therefore, the primary objective for this study is to identify the demographic, psychological and behavioural factors associated with drinking above the current national alcohol guideline recommendations among Australian midlife men. This study will also explore a secondary objective, examining whether these factors differ between younger and older midlife men to obtain a greater understanding of whether specific factors influence risky drinking at earlier or later stages of midlife.

## 2 Methods

### 2.1 Data Source Used

This study employed secondary data analysis of the 2019 and 2022/23 waves of the NDSHS [15, 46]. The NDSHS is Australia's primary source of data on alcohol and other drugs, conducted every 3 years and employs self‐report questionnaires to random households across Australian states and territories. Selected households would then issue the survey to the household member aged 14 years or older who recently celebrated their birthday. The selected respondent would then complete the survey via the delivered form or online with the option to opt‐out entirely at any stage. The NDSHS assesses personal alcohol, tobacco, e‐cigarette and other drug use in addition to opinions towards policy change, demographic, psychological and behavioural factors. The 2019 wave of the NDSHS comprised 22,272 completed responses (49.0% response rate) [46] while the 2022/23 wave comprised 21,661 completed responses (43.9% response rate) [15]. For this study, the two most recent waves were combined to enhance analytic power for all analyses undertaken. Unless specifically stated, all analyses undertaken incorporate sampling weights provided by the data custodians to ensure estimates are broadly representative of the Australian population (full details reported elsewhere) [15, 46].

### 2.2 Measures

#### 2.2.1 Sample Demographic

Samples considered were restricted to those who met two criteria: reported being ‘male’ and reported being midlife aged, defined as 30–59 years for this study. Respondents were asked ‘*What is your sex*?’ in the 2019 wave [46] and ‘*How do you describe your gender*?’ in the 2022/23 wave [15]. For both waves, respondents were asked ‘*What is your current age*?’. The samples who were aged 30–59 were included (*n* = 9006). The included samples were then split into two midlife groups. Those aged 30–44 years were considered ‘Younger Middle Adults’ (YMA) (*n* = 4516), and 45–59 years were considered ‘Older Middle Adults’ (OMA) (*n* = 4490). Similar age groups have previously been used in the Ten to Men Australian longitudinal study [24]. Only responses from people who met the criteria of ‘midlife men’ and responded to all factors of interest below were included (*n* = 6471). A detailed list of samples excluded from the baseline sample can be found on Supplementary Document S1.

#### 2.2.2 Outcome Variable

Respondents were asked various questions exploring alcohol consumption, including ‘In the last 12 months, how often did you have an alcoholic drink of any kind?’. For those responding that they had consumed alcohol, further questions inquired about how frequent alcohol was consumed during single‐occasion drinking sessions (20 or more, 11–19, 7–10, 5–6, 3–4, 1–2, less than 1, none) and drinking throughout the week (every day, 5–6 days a week, 3–4 days a week, 1–2 days a week, 2–3 days a month, about 1 day a month, less often, never). Annual estimates of alcohol consumed were reflected based on responses of the most recent iteration of the National Health and Medical Research Council's (NHMRC) Alcohol Guideline 1: ‘Reducing the risk of alcohol‐related harm for adults’ in 2020 [47]. That is,

‘To reduce the risk of harm from alcohol‐related disease or injury, healthy men and women should drink no more than 10 standard drinks a week and no more than 4 standard drinks on any one day’ [47] (p. 2)

For the 2019 wave, a later update recoded responses relating to alcohol consumption to reflect the changes made by the NHMRC to Alcohol Guideline 1 in 2020 [46]. Drinking in excess of these guidelines was considered if the respondent reported alcohol consumption that exceeded more than four standard drinks on any occasion in the last month, or more than 10 standard drinks within a week in the last 12 months. Respondents were then split into two dichotomous groups based on responses to the alcohol‐related questions: No (abstained from alcohol OR did not exceed guidelines) and Yes (exceeded guidelines). Those who responded ‘*yes*’ were classified as having exceeded national alcohol guidelines and have engaged in ‘*risky drinking’* here throughout.

#### 2.2.3 Variables of Interest

Modifiable risky behaviours included responses to use of tobacco products (current smoking; abstainer or former smoker) and illicit/unprescribed drugs in the last 12 months (yes to at least one substance; no drug use). The smoking variable only considered responses for tobacco, manufactured cigarette use, cigars and pipes but did not include vaping products.

Psychological health factors included the Kessler Psychological Distress Scale‐10 (K10) [48], exploring self‐reported psychological distress scores (low‐moderate distress [*scores between 10–21*]; high‐very high distress [*scores between 22–50*]) and having a formal diagnosis or treatment of mental health conditions including anxiety and depression (yes to at least one; no).

Demographic variables of interest included: socio‐economic status (SES) based on the Index of Relative Socio‐Economic Advantage and Disadvantage [49] 1–5 quintile structure (classifications ranging from ‘1’ [most disadvantaged] to ‘5’ [most advantaged]), rurality classified by the Australian Statistical Geography Standard 2016 [50] (Metropolitan [described as ‘major cities’]; Rural/Remote/Regional [regional included both ‘inner’ and ‘outer’ zones]), marital status (Never Married; Divorced/Widowed/Separated; Married/De Facto), dependent children living in household (no children; 1+ children), peak education attainment (did not finish secondary school; completed secondary school; completed diploma/cert III +; completed bachelors' degree +), occupational groups based on the Australian and New Zealand Standard Classification of Occupations [51] (managers; professionals; tech and trades; skilled; unskilled) and household incomes (in Australian Dollars) (don't know/prefer not to say; low income [$999 or less per week]; middle income [$1000–1999 per week]; high income [$2000 or more per week]). Areas defined using the Index of Relative Socio‐Economic Advantage and Disadvantage quintile classifications are based on factors including access to health services, employment and various social and essential resources [49]. Categorisation for occupational groups were adopted from another health study that examined the prevalence of tobacco use in Australian occupations; ‘Managers’, ‘Professionals’, ‘Tech and Trades’, ‘Skilled Workers (Community and Personal Service, Clerical and Administrative and Machine Operators and Drivers)’ and ‘Unskilled Workers (Sales Workers, and Labourers)’ [52].

### 2.3 Analytic Strategy

Analyses were undertaken in IBM SPSS Statistics v29 and STATA v18. Relative weighting was applied for all analyses conducted and statistical significance was considered if *p* < 0.05. SPSS analyses were conducted for descriptive statistics through crosstabulation and chi‐square analyses to test for significant differences in the prevalence of midlife men who reported risky drinking and the selected demographic, psychological and behavioural variables. STATA analyses employed ‘svy’ commands [53] to adjust for the complex survey sampling and applied weights across a series of multivariable logistic regression models. First, an initial model containing all variables of interest was conducted to examine the likelihood of risky drinking in the presence of all variables against their respective reference categories. Following this, a separate series of 11 individual interaction models were conducted to examine interactions between age group and each independent variable individually to assess whether the effects of these variables on risky drinking differed by age. Full interactions for variables that were significant in these individual models were then included in the final model. STATA commands ‘margins’ and ‘marginsplot’ were employed to interpret all individual interactions visually. As this study is explorative, the analytical processes were not pre‐registered.

## 3 Results

Table 1 presents descriptive statistics for the sample, along with the prevalence of risky drinking among midlife men by each demographic, psychological and behavioural variable. Overall risky drinking in this study was reported by 42.9% of midlife men. Engagement in risky drinking appeared most prevalent among midlife men who also engaged in other modifiable risky behaviours, including those who reported currently smoking tobacco (58.9%, 95% confidence interval [54.7–62.9]) and using illicit drugs or non‐prescribed use of medications (69.2% [65.6–72.5]). Those who resided in areas classified as quintile 2 SES (slightly disadvantaged areas) (45.0% [41.0–49.1]) and lived in rural/remote/regional locations (52.6% [49.3–55.8]) reported higher prevalence of risky drinking among Australian midlife men. Considering psychological factors, the prevalence of risky drinking was highest among midlife men reporting high‐very high psychological distress scores (50.5% [45.4–55.6]). Other factors with greater prevalence of risky drinking included midlife men who reported being divorced/widowed/separated (48.0% [41.5–54.4]), achieving a peak educational attainment of Diploma/Cert III + (49.6% [46.7–52.5]), worked an occupation classified as managerial (48.9% [44.8–53.1]) or Tech/Trade (50.4% [46.7–54.2]) and lived in a household that earned $2000 or more per week (49.5% [47.0–52.1]).

**TABLE 1:** Demographic information for midlife men aged 30–59 years who reported drinking in excess of National Health and Medical Research Council 2020 Alcohol Guideline 1 in 2019 and 2022/23 National Drug Strategy Household Survey waves.

Factors	Demographic	*n* (%)	Risky drinking prevalence (95% CI)	Sig
			Weighted = 42.9%	
Midlife age groups	Younger middle adults	3311 (51.4%)	43.3% (40.8–45.7)	χ2 (1, 6408) = 0.28, *p* = 0.60
	Older middle adults	3160 (48.6%)	42.6% (40.2–45.1)	
Smoker status	Former/non‐smoker	5424 (84.3%)	40.0% (38.1–41.9)	χ2 (1, 6408) = 123.37, *p* < 0.001**
	Currently smoking	1047 (15.7%)	58.9% (54.7–62.9)	
Illicit drug use (last 12 months)	Not using drugs (last 12 months)	5075 (79.8%)	36.3% (34.4–38.2)	χ2 (1, 6408) = 455.49, *p* < 0.001**
	Used drugs (last 12 months)	1396 (20.2%)	69.2% (65.6–72.5)	
Marital status	Never married	1126 (14.5%)	41.6% (37.1–46.1)	χ2 (2, 6408) = 6.36, *p* = 0.04*
	Divorced/widowed/separated	619 (8.2%)	48.0% (41.5–54.4)	
	Married/de facto	4726 (77.3%)	42.7% (40.7–44.7)	
Peak educational attainment	Did not finish high school	743 (11.0%)	46.6% (41.7–51.5)	χ2 (3, 6408) = 89.19, *p* < 0.001**
	Completed year 12	885 (13.6%)	42.2% (37.2–47.4)	
	Completed Diploma/Cert III+	2187 (34.7%)	49.6% (46.7–52.5)	
	Completed Bachelors' Degree+	2656 (40.7%)	36.5% (33.8–39.2)	
Socio‐economic status (IRSAD)	1—Most disadvantaged	919 (15.9%)	38.3% (34.0–42.7)	χ2 (4, 6408) = 16.48, *p* = 0.002*
	2—Slightly disadvantaged	1110 (18.2%)	45.0% (41.0–49.1)	
	3—Neutral	1306 (21.5%)	44.9% (41.0–48.8)	
	4—Slightly advantaged	1527 (22.9%)	41.2% (37.5–45.0)	
	5—Most advantaged	1609 (21.6%)	44.5% (40.7–48.4)	
Rurality (ASGS)	Metropolitan	4704 (75.3%)	39.8% (37.7–41.9)	χ2 (1, 6408) = 79.38, *p* < 0.001**
	Rural/regional/remote	1767 (24.7%)	52.6% (49.3–55.8)	
Dependent children in HH	No dependent children in HH	3032 (41.8%)	43.4% (40.8–46.0)	χ2 (1, 6408) = 0.42, *p* = 0.52
	1+ dependent children in HH	3439 (58.2%)	42.6% (40.3–45.0)	
Psychological distress (K10)	Low‐Mod Psych Dist K10 Score	5644 (87.8%)	41.9% (40.0–43.8)	χ2 (1, 6408) = 20.80, *p* < 0.001**
	High‐VHigh Psych Dist K10 Score	827 (12.2%)	50.5% (45.4–55.6)	
Diagnosis and treatment of MH	No diagnosis/treatment of MH	5543 (86.3%)	42.5% (40.6–44.4)	χ2 (1, 6408) = 2.85, *p* = 0.09
	Yes diagnosis/treatment of MH	928 (13.7%)	45.6% (40.8–50.4)	
Occupational group (ANZSCO)	Manager occupation	1212 (17.7%)	48.9% (44.8–53.1)	χ2 (4, 6408) = 75.37, *p* < 0.001**
	Professional occupation	1972 (29.0%)	38.6% (35.5–41.8)	
	Tech and trade occupation	1223 (19.9%)	50.4% (46.7–54.2)	
	Skilled occupation	1388 (22.5%)	38.0% (34.4–41.8)	
	Unskilled occupation	676 (10.9%)	41.2% (36.3–46.3)	
Household income	Don't know/prefer not to say HHI	838 (14.1%)	27.8% (24.0–31.8)	χ2 (3, 6408) = 164.00, *p* < 0.001**
	Low—$999 or less per week HHI	622 (8.8%)	33.6% (28.1–39.6)	
	Mid—$1000‐$1999 per week HHI	1739 (26.5%)	41.5% (38.2–44.9)	
	High—$2000 or more per week HHI	3272 (50.5%)	49.5% (47.0–52.1)	

*Note:* Raw samples and relative weighted percentage and significance values were reported. Raw *n* for “Yes” Response = 2,800, weighted *n* = 2751. Raw *n* for “No” Response = 3,671, weighted *n* = 3657. Raw *n* = 6471 (42.9% reported drinking exceeding guideline 1).

*Abbreviations:* ANZSCO, Australian and New Zealand Standard Classification of Occupations; ASGS, Australian Statistical Geography Standard 2016; CI, confidence interval; HH, household; HHI, household income; IRSAD, Index of Relative Socio‐Economic Advantage and Disadvantage; MH, mental health; Psych Dist K10, Kessler Psychological Distress Scale (K10).

*Category is significant as *p* < 0.05, **category is highly significant as *p* < 0.001.

To examine the relationship between socio‐demographic factors and risky drinking across the whole sample, an initial multivariable logistic regression model was created to explore the likelihood of engagement in risky drinking among Australian midlife men (see Table 2). This regression model found that those currently smoking, using illicit or non‐medical use of drugs, residing in locations within classification 3 SES (neutral areas) and 5 (most advantaged areas), living in rural/remote/regional locations, high‐very high psychological distress scores, working in a managerial or Tech/Trade occupation and living in households that earned $2000 or more per week were significantly more likely than their respective reference categories to engage in risky drinking. In contrast, those who reported attaining a Bachelors' Degree +, and either did not know or disclose their household earnings or reported household earnings of $999 and lower per week were significantly less likely to engage in risky drinking compared to their respective reference categories.

**TABLE 2:** Initial regression model for drinking in excess of National Health and Medical Research Council 2020 Alcohol Guidelines (compared to not drinking in excess) among all midlife men aged 30–59 years from 2019 and 2022/23 National Drug Strategy Household Survey waves.

Variables and categories	OR	95% CI		*p*
		Lower	Upper	
Age groups (RC: Younger middle adults)				
Older middle adults	1.00	0.85	1.17	0.99
Smoker status (RC: Former/non‐smoker)				
Currently smoking	1.70	1.35	2.14	< 0.001**
Used illicit drugs (last 12 months) (RC: Non‐user)				
Used last 12 months	3.54	2.92	4.31	< 0.001**
Marital status (RC: Single/Never married)				
Divorced/widowed/separated	1.22	0.87	1.73	0.25
Married/de facto	1.16	0.89	1.50	0.27
Educational attainment (RC: DNF high school)				
Completed year 12	0.88	0.64	1.22	0.46
Diploma/Cert III+	0.98	0.76	1.27	0.90
Bachelor's Degree+	0.61	0.46	0.81	0.001*
SES (IRSAD) (RC: 1 – Most disadvantaged)				
2—Slightly disadvantaged	1.19	0.92	1.55	0.19
3—Neutral	1.37	1.05	1.78	0.02*
4—Slightly advantaged	1.30	0.99	1.72	0.06
5—Most advantaged	1.53	1.15	2.03	0.003*
ASGS (Rurality) (RC: Metropolitan)				
Rural/remote/regional	1.76	1.48	2.11	< 0.001**
Dep child in household (RC: No children)				
1+ children	0.95	0.81	1.12	0.58
Psychological distress (K10) (RC: Low‐Mod scores)				
High‐Very high scores	1.37	1.05	1.79	0.02*
Diagnosis or treatment MH (RC: No)				
Yes	0.90	0.70	1.16	0.42
Occupational group (RC: Skilled professions)				
Managers	1.48	1.14	1.91	0.003*
Professionals	1.11	0.87	1.43	0.40
Tech and trades	1.48	1.17	1.87	0.001*
Unskilled professions	1.18	0.89	1.56	0.25
Household income (RC: Mid ($1000–1999 per week))				
Don't know/Prefer not to say	0.58	0.45	0.75	< 0.001**
Low ($999 or less per week)	0.64	0.45	0.91	0.01*
High ($2000 or more per week)	1.70	1.41	2.06	< 0.001**

*Note:* Raw *n* = 6,471. Relative weighted *n* = 6,408, absolute weighted *n* = 7,243,226.*Category is significant as *p* < 0.005, **Category is highly significant as *p* < 0.001.

*Abbreviations:* ASGS, Australian Statistical Geography Standard 2016; CI, confidence interval; Dep, dependent; DNF, did not finish; IRSAD, Index of Relative Socio‐Economic Advantage and Disadvantage; MH, mental health; OR, odds ratio; RC, reference category; SES, socio‐economic status.

**FIGURE 1:**
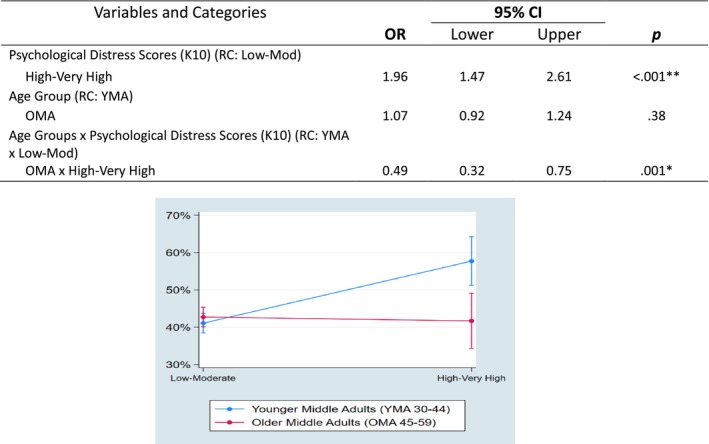
Interactions and plot between age groups and psychological distress scores based on Kessler 10 scale. Abbreviations: CI, confidence interval; OR, odds ratio; RC, reference category.

**FIGURE 2:**
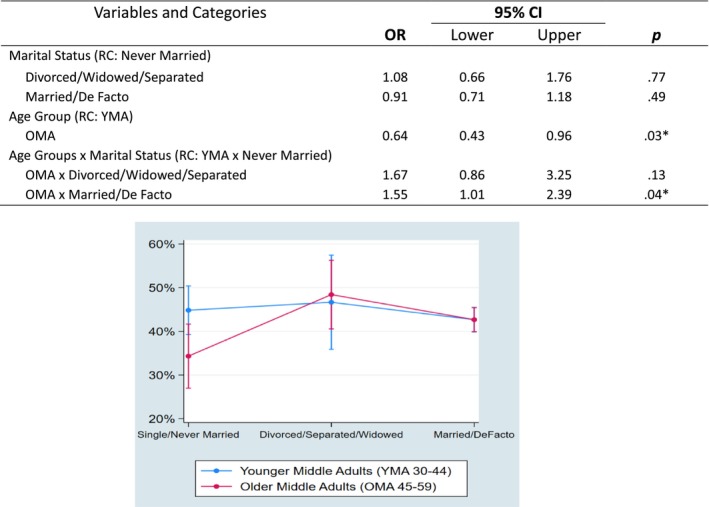
Interactions and plot between age groups and marital status. Abbreviations: CI, confidence interval; OR, odds ratio; RC, reference category.

The next set of regression analyses examined interactions between two midlife age groups and the factors of interest individually to assess whether the relationships between the independent variables and risky drinking varied by age groups. A total of 11 individual interaction models were conducted, separately interacting each factor with age group. Of these, two individual regression models produced significant interactions between midlife age groups and the factors of interest (non‐significant findings shown in Supplementary Document S1). The first significant interaction was between age groups and psychological distress scores. YMA who reported high–very high psychological distress scores were significantly more likely to report risky drinking than YMA who scored low–moderate psychological distress scores. For OMA, the likelihood of risky drinking did not differ with the level of psychological distress (see Figure 1). Marital status was the other significant interaction with age groups, with OMA who reported current relationships as married/de facto being significantly more likely to engage in risky drinking when compared with OMA who had never married. For YMA, differences between marital status and age groups did not differ (see Figure 2).

The final regression model added these two significant interactions into the initial model (see Table 3). With inclusion of these significant interactions, only the interaction between YMA and high‐very high psychological distress scores remained significant.

**TABLE 3:** Final regression model for drinking in excess of National Health and Medical Research Council 2020 Alcohol Guidelines (compared to not drinking in excess) among all midlife men aged 30–59 years from 2019 and 2022/23 National Drug Strategy Household Survey waves.

Variables and categories	OR	95% CI		*p*
		Lower	Upper	
Age groups (RC: Younger middle adults)				
Older middle adults	0.83	0.54	1.29	0.42
Smoker status (RC: Former/Non‐smoker)				
Currently smoking	1.72	1.38	2.15	< 0.001**
Used illicit drugs (last 12 months) (RC: Non‐user)				
Used last 12 months	3.58	2.95	4.34	< 0.001**
Marital status (RC: Never married)				
Divorced/widowed/separated	0.83	0.46	1.51	0.54
Married/de facto	1.07	0.79	1.45	0.65
Age groups × Marital status (RC: YMA × Never married)				
OMA × Divorced/widowed/separated^a^	2.02	0.97	4.23	0.06
OMA × Married/de facto	1.32	0.83	2.09	0.24
Educational attainment (RC: DNF high school)				
Completed year 12	0.87	0.63	1.19	0.38
Diploma/Cert III+	0.96	0.74	1.24	0.77
Bachelor's Degree+	0.59	0.44	0.79	< 0.001**
IRSAD (SES) (RC: 1—Most disadvantaged)				
2—Slightly disadvantaged	1.23	0.94	1.60	0.12
3—Neutral	1.38	1.06	1.79	0.02*
4—Slightly advantaged	1.33	1.01	1.77	0.04*
5—Most advantaged	1.56	1.17	2.06	0.002*
ASGS (Rurality) (RC: Metropolitan)				
Rural/remote/regional	1.76	1.47	2.10	< 0.001**
Dep child in household (RC: No children)				
1+ Children	0.97	0.82	1.14	0.72
Psychological Distress (K10) (RC: Low‐Mod Scores)				
High‐Very high scores	1.92	1.37	2.69	< 0.001**
Age groups × Psychological Distress (K10) (RC: YMA × Low‐Mod Scores)				
OMA × Low‐Mod Scores^a^	0.47	0.29	0.76	0.002*
Diagnosis or treatment MH (RC: No)				
Yes	0.92	0.72	1.17	0.51
Occupational group (RC: Skilled professions)				
Managers	1.44	1.12	1.85	0.005*
Professionals	1.10	0.85	1.41	0.46
Tech and trades	1.45	1.15	1.83	0.002*
Unskilled professions	1.15	0.87	1.53	0.32
Household ncome (RC: Mid [$1000–1999 per week])				
Don't know/prefer not to say	0.59	0.46	0.76	< 0.001**
Low ($999 or less per week)	0.66	0.46	0.94	0.02*
High ($2000 or more per week)	1.72	1.42	2.08	< 0.001**

*Note:* Raw *n* = 6471. Relative weighted *N* = 6408, absolute weighted *N* = 7,243,226. *Category is significant as *p* < 0.005, **Category is highly significant as *p* < 0.001.

*Abbreviations:* ASGS, Australian Statistical Geography Standard 2016; CI, confidence interval; Dep, dependent; DNF, did not finish; IRSAD, Index of Relative Socio‐Economic Advantage and Disadvantage; MH, mental health; OMA, Older Middle Adults; OR, odds ratio; RC, reference category; SES, socio‐economic status; YMA, Younger Middle Adults.

^a^ OMA × Never Married and OMA × High‐Very High Scores omitted due to multicollinearity.

To assess the impact of dichotomising risky drinking based on the NHMRC alcohol guidelines, a sensitivity analysis using higher thresholds was conducted. These higher thresholds were the equivalent of double or higher than the NHMRC guidelines (defined as the ‘exceeded ‐ high risk’ group). Results from this analysis and full details around the creation of this variable are presented in Supplementary Document 2. Findings from these analyses were broadly similar to those presented here, with the only major shift being present among age groups, where OMA men were significantly more likely to drink at ‘exceeded ‐ high risk’ levels when compared to YMA.

## 4 Discussion

In Australia, men are more likely to engage in risky drinking in excess of Australia's Alcohol Guidelines when compared to women [16, 25], with trends for alcohol consumption among midlife men exclusively continuing to remain stable [24, 25, 54]. This study differed from existing studies by examining various demographic, psychological and behavioural factors and their potential association with risky drinking among midlife men, in addition to exploring whether different stages of midlife played a role. These analyses intended to support and inform future public health policy and health promotion efforts for Australian midlife men, a group currently underrepresented in the literature [34, 41]. Overall, this study found 42.9% of Australian midlife men aged 30–59 years consumed alcohol at levels that exceeded national alcohol guideline recommendations. Other Australian studies, such as the Ten to Men study, have identified varying trends differing from this study's findings, summarising that approximately 3 in 10 midlife aged men bordered risky drinking thresholds, with recommendations of monitoring this group of men for any future changes in drinking trajectories [24]. However, that study utilised the Alcohol Use Disorders Identification Test to score and measure risky drinking, whereas our study used a definition of risky drinking that aligned with Australia's current alcohol guideline recommendations [47].

This study found that higher psychological distress was associated with a greater likelihood of engagement in risky drinking among YMA men. This finding is consistent with existing studies that highlight increased stress levels as a risk factor for risky drinking among specifically midlife populations. Factors including stressful workdays [35, 36, 42, 43, 55, 56], financial hardships [36, 57] and mental health factors (including pre‐existing depression and anxiety) [40, 58] are just some examples potentially contributing to this link. Other studies exploring midlife populations highlight alcohol use as a means of minimising feelings of stress and anxiety, while maximising benefits of relaxation, suggesting that the visibility of alcohol, typically within the home, acts as a viable option to unwind from negative thoughts [42, 43, 59]. In attempts to understand these psychological factors thoroughly, these studies employed qualitative approaches, focusing on samples aged 40 years and above and were not exclusive to midlife men. When compared to the OMA men from this study, despite being a similar age group to the previously mentioned studies, no association between psychological distress and risky drinking was identified, suggesting that potential cohort differences may be present. While this study only examined psychological distress through one self‐report screening tool [48], further exploration into the causes and experiences of psychological distress among midlife men, and its direct and indirect relationships with alcohol use, is warranted.

In addition to psychological distress, this study identified various other factors that were significantly associated with risky drinking and midlife men. Firstly, drinking in excess of guidelines was significantly associated with other modifiable risky behaviours, including current use of other substances (e.g., tobacco and illicit/non‐medicinal drugs). International studies have substantiated this link among all age groups [24, 40, 60, 61], suggesting that the use of alcohol and other substances may act as a coping mechanism to alleviate poor mental health and wellbeing, including stress, depression and anxiety [62, 63, 64]. While existing studies have previously highlighted that typical same‐day poly‐use combinations of alcohol/tobacco, and alcohol/cannabis occurs predominately among young adults [65, 66], findings from our study suggest that this link continues to remain prominent upon transitioning into midlife. The poly‐use of alcohol alongside other recreational substances, and their synergistic detrimental effects on health and wellbeing justifies increased health promotion efforts to target these behaviours (either in isolation or simultaneously) [63, 65, 67]. There is evidence for addressing poly‐use of substances simultaneously, albeit with a substance dependent population. A meta‐analysis by Prochaska and colleagues [68] found that smoking cessation interventions provided during treatment was associated with a 25% increased likelihood of long‐term abstinence from alcohol and other drugs. In addition, implementation of other strategies including Australia's National Tobacco Control Strategy 2023–2030 [69] and Australia's National Alcohol Strategy 2019–2028 [70] would make vital progress for this target group. Furthermore, considerations recommended by the World Health Organization's ‘SAFER strategies’ may also play a critical role, in particular implementing universal screening programs to primary health care providers, which could lead to the triaging individuals in this category to tailored programs and interventions [71].

Rurality was found to be strongly associated with risky drinking, remaining consistent with existing research [10, 24, 72]. Previous studies have discussed reasons for this including social norms [72, 73], increased attendances to close‐knit community groups such as sports clubs [74] and reduced access to counselling, health and support facilities [72, 75]. A report by Smith and colleagues [76, 77] highlights the increased rates of alcohol consumption among populations living in Australian rural and regional zones. While some rural health workforce agencies are receiving some funding, certain specialist and non‐clinical health promotion professionals feel unsupported and receive less external aid when compared to their metropolitan counterparts, deterring the longevity of these health services [77]. In addition, while community groups are often common ground among rural and regional locations, community‐wide approaches for health promotion among younger cohorts have demonstrated effectiveness in addressing and deterring alcohol cultures (including normalising or encouraging risky drinking), incentivising longer‐term approaches to be developed and implemented among varying age groups [78]. Targeting non‐metropolitan midlife men for early intervention and gaining timely access to treatment for potential risky alcohol use is vital for advancing positive outcomes for men in rural and remote Australia, and more specifically, addressing the structural barriers to healthcare services and the impacts of isolated employment on mental health and wellbeing.

Many studies have emphasised factors relating to educational qualifications and occupations as strong predictors of health outcomes [55, 61, 79, 80]. This study builds on findings from these studies highlighting that risky drinking among midlife men is more prevalent in lower educational groups, such as those who did not complete secondary schooling and those who have completed non‐university based tertiary education. Prior links between education and occupation have frequently been identified, suggesting that existing prerequisites for specific occupations are required to work certain roles. One example of this involves greater prevalence in risky drinking among those working tech or trade occupations, another finding in our study that has remained consistent with others. A systematic review conducted by Roche and colleagues [55] highlighted that the nature of tech and trade occupations, alongside the roles, responsibilities and workplace cultures, are more likely to attract men. An international study extends on this, highlighting high‐intensity blue‐collar occupations having greater susceptibility to such risky drinking patterns [80] with factors including strains on mental health, stress and burnout being contributors to such drinking cultures [55, 80, 81]. In contrast, a link in significantly lower likelihood of risky drinking was uncovered between attaining a bachelor's degree and working in either professional or skilled occupations. The nature of these roles requires specific educational qualifications to act as a proxy; however, the requirement of advanced skillsets within occupations of these classifications potentially deters risky drinking behaviours from occurring across midlife populations [80]. This is speculated to be a result of having greater understandings around the harms and risks associated with alcohol use. Prior calls for workplace health promotion and intervention programs tailored to specific occupations continue to remain relevant, with aims of achieving positive lifestyle outcomes [55, 80].

### 4.1 Limitations and Future Considerations

The NDSHS has consistently been reliable in examining various alcohol and drug‐related data across various Australian quantitative designs [26, 36, 54, 82, 83]. However, there are some limitations. The study is cross‐sectional in nature and therefore drawing causations from findings in this study cannot be made. In addition, the NDSHS collects self‐reported responses for questions relating to alcohol and other substances, which may underestimate consumption, particularly among respondents who engage in heavier drinking [84]. We have also included suspected dependent drinkers in this analysis, which is a limitation of the NDSHS data being unable to directly differentiate those who exceeded guidelines once from those who have exceeded guidelines more often. Finally, this study combined data from the two most recent waves of the NDSHS, where one wave comprised results 2 years post COVID‐19 pandemic. While a decline in risky drinking was identified in the 2022/23 sample compared to the 2019 (pre COVID‐19) wave, we are unable to determine within this dataset whether COVID‐19 or elements of the pandemic including lockdowns or restrictions were factors for this declining trend.

Future investigations may look to explore specific risk factors among emerging and current midlife men through longitudinal data to explore possible transitional influence leading to risky drinking among this group. In addition, this approach would enable these factors to be triangulated, identifying opportunities to tailor appropriate health promotion strategies towards the identified factors with the intent of positively impacting the health and wellbeing of midlife men. There is currently limited literature investigating the motivations of specific midlife men and risky drinking within Australia. While this study highlighted factors linking risky drinking with midlife men through a single‐gender focus, there is an opportunity to investigate why this occurs through a qualitative design to examine whether these are synergistic with, or differ from, those of midlife women and create opportunities for the development of single‐gender and gender neutral health promotion options that address risky drinking.

## 5 Conclusion

While risky drinking among Australian men continues to decline, this study has revealed that a greater proportion of Australian midlife men are currently drinking at levels that exceed national alcohol guideline recommendations and therefore at increased risk of health harms. Findings suggest that there may be substantial benefits to targeting preventive health efforts among specific groups of men engaging with risky drinking, including those reporting higher levels of psychological distress, marital status and among those residing outside of metropolitan areas. Our findings encourage further investigations predominately focusing on midlife men, and more specifically, possible routine alcohol consumption throughout the week. With continued declines of risky drinking identified among younger men, future research, intervention and public health policy efforts targeting midlife men may aid in continuing this trend in alcohol consumption.

## Author Contributions

Conceptualisation: Stefano Zaccagnini, Ashlea Bartram, Michael Livingston, James A. Smith, Nataly Bovopoulos, Jacqueline Bowden. Data recoding: Stefano Zaccagnini, Michael Livingston. Analysis of data: Stefano Zaccagnini. Study methodology: Stefano Zaccagnini, Ashlea Bartram, Michael Livingston, James A. Smith, Nataly Bovopoulos, Jacqueline Bowden. Writing of original draft: Stefano Zaccagnini. Reviewing and editing of drafts: Stefano Zaccagnini, Ashlea Bartram, Michael Livingston, James A. Smith, Nataly Bovopoulos, Jacqueline Bowden.

## Funding

This work was supported by the Flinders University and the Alcohol and Drug Foundation.

## Conflicts of Interest

A.B., M.L. and J.B. receive funding from the Australian Department of Health, Disability and Ageing to support research regarding alcohol and other drugs. M.L. is also supported by the Australian Research Council (ARC) (FT210100656). J.A.S. receives funding for alcohol‐related research from the ARC (LP180100701), NT Department of Health and NT Community Benefit Fund. S.Z. receives co‐funding from Flinders University and Alcohol and Drug Foundation via the Flinders University PhD Enterprise Scholarship. The authors have no conflicts of interest to declare.

## Supporting information


**Table S1:** Baseline responses for respondents' drinking above or below the NHMRC alcohol guidelines that were classified as “Midlife Men”. Raw *n* = 6439.
**Table S2:** Descriptive Statistics for midlife men reporting drinking volume for ALL Midlife Men (weighted). Raw *n* = 6439.
**Table S3:** Initial regression model for drinking in the ‘exceeded ‐ high risk’ group of NHMRC 2020 Guidelines (compared to drinking in the ‘did not exceed’ group and ‘exceeded’ group) based on volume estimates among all midlife men aged 30–59 years from 2019 and 2022/23 NDSHS waves.

**Data S1:** Summary of respondents excluded due to incomplete/uninterpretable/invalid responses (values based on raw *n*).

## Data Availability

The data that support the findings of this study are available from National Drug Strategy Household Survey. Restrictions apply to the availability of these data, which were used under license for this study. Data are available from the author(s) with the permission of National Drug Strategy Household Survey.
