# Supplementary material for: Risky Drinking in Midlife Men: Insights From Australia's National Drug Strategy Household Survey
Source: Drug Alcohol Rev. 2026 Apr 13;45(4):e70149. doi: 10.1111/dar.70149 (PMC13077182; doi:10.1111/dar.70149)
Supplement: Supplementary file 1 — Table S1: Baseline responses for respondents' drinking above or below the NHMRC alcohol guidelines that were classified as “Midlife Men”. Raw n = 6439. Table S2: Descriptive Statistics for midlife men reporting drinking volume for ALL Midlife Men (weighted). Raw n = 6439. Table S3: Initial regression model for drinking in the ‘exceeded ‐ high risk’ group of NHMRC 2020 Guidelines (compared to drinking in the ‘did not exceed’ group and ‘exceeded’ group) based on volume estimates among all midlife men aged 30–59 years from 2019 and 2022/23 NDSHS waves. [file DAR-45-0-s002.docx]

| **Supplementary Table 1**: Baseline responses for respondents’ drinking above or below the NHMRC alcohol guidelines that were classified as “Midlife Men”. Raw n = 6,439 | | | |
| --- | --- | --- | --- |
|  | 2019 wave | 2022/23 wave | Total |
| No – Did not exceed 2020 NHMRC Guidelines | 1,641 (54.5%) | 2,030 (59.2%)* | 3,671 (57%) |
| Yes – Exceeded 2020 NHMRC Guidelines | 1,371 (45.5%)* | 1,397 (40.8%) | 2,768 (43%) |
| **Note:** * = differences between waves were significant | | | |

When focusing predominately on midlife aged men (aged 30-59 years) that participated in the 2019 and 2022/23 waves of the National Drug Strategy Household Survey (NDSHS), there was a significant difference in risky drinking behaviours between 2019 (more likely to exceed guidelines) than 2022/23 (less likely to exceed guidelines).

| **Supplementary Table 2**: Descriptive Statistics for midlife men reporting drinking volume for ALL Midlife Men (weighted). Raw n = 6439 | | | | | |
| --- | --- | --- | --- | --- | --- |
|  | **Demographic** | **Did not exceed** | **Exceeded** | **Exceed - High Risk** | **Total** |
| **Response Category** |  | 4600 (72.2%) | 1021 (16%) | 754 (11.8%) | 6375 |
|  |  |  |  |  |  |
| **Midlife Group** | YMA | 2433 (74.2%)* | 503 (15.4%) | 340 (10.4%) | 3277 (51.4%) |
|  | OMA | 2167 (69.9%) | 517 (16.7%) | 414 (13.4%)* | 3098 (48.6%) |
|  |  |  |  |  |  |
| **Smoker Status** | Non/Former Smoker | 4045 (75.2%)* | 846 (15.7%) | 489 (9.1%) | 5380 (84.4%) |
|  | Currently Smoking | 555 (55.8%) | 175 (17.6%) | 265 (26.6%)* | 995 (15.6%) |
|  |  |  |  |  |  |
| **Drug Use (12 months)** | No Illicit Drug Use | 3920 (76.9%)* | 730 (14.3%) | 446 (8.8%) | 5096 (79.9%) |
|  | Using Illicit Drugs | 680 (53.2%) | 291 (22.8%)* | 308 (24.1%)* | 1279 (20.1%) |
|  |  |  |  |  |  |
| **Marital Status** | Single/Never Married | 698 (75.3%)* | 92 (9.9%) | 137 (14.8%)* | 927 (14.5%) |
|  | Divorced/Widow/Sep | 339 (65.7%) | 77 (14.9%) | 100 (19.4%)* | 516 (8.1%) |
|  | Married/DeFacto | 3563 (72.2%) | 852 (17.3%)* | 517 (10.5%) | 4932 (77.4%) |
|  |  |  |  |  |  |
| **Peak Education** | DNF Year 12 | 477 (67.6%) | 92 (13%) | 137 (19.4%)* | 706 (11.1%) |
|  | Completed Year 12 | 630 (72.6%) | 140 (16.1%) | 98 (11.3%) | 868 (13.6%) |
|  | Completed Dip/Cert III | 1486 (67.4%) | 388 (17.6%)* | 330 (15%)* | 2204 (34.6%) |
|  | Completed Bach+ | 2007 (77.3%)* | 401 (15.4%) | 189 (7.3%) | 2597 (40.7%) |
|  |  |  |  |  |  |
| **SES (SEIFA)** | 1 – M. Disadvantaged | 781 (77%)* | 111 (10.9%) | 122 (12%) | 1014 (15.9%) |
|  | 2 – S. Disadvantaged | 805 (69.3%) | 178 (15.3%) | 178 (15.3%)* | 1161 (18.2%) |
|  | 3 – Neutral | 967 (70.7%) | 226 (16.5%) | 174 (12.7%) | 1367 (21.4%) |
|  | 4 – S. Advantaged | 1068 (73.3%) | 243 (16.7%) | 147 (10.1%) | 1458 (22.9%) |
|  | 5 – M. Advantaged | 979 (71.3%) | 262 (19.1%)* | 132 (9.6%) | 1373 (21.5%) |
|  |  |  |  |  |  |
| **Rurality (ASGS)** | Metropolitan | 3599 (75.1%)* | 705 (14.7%) | 491 (10.2%) | 4795 (75.2%) |
|  | Rural/Regional/Remote | 1001 (63.4%) | 316 (20%)* | 263 (16.6%)* | 1580 (24.8%) |
|  |  |  |  |  |  |
| **Dep Children** | No Children | 1896 (71.2%) | 406 (15.2%) | 361 (13.6%)* | 2663 (41.8%) |
|  | 1+ Children | 2704 (72.8%) | 615 (16.6%) | 393 (10.6%) | 3712 (58.2%) |
|  |  |  |  |  |  |
| **Psych Distress (K10)** | Low-Moderate Scores | 4069 (72.7%)* | 893 (15.9%) | 638 (11.4%) | 5600 (87.9%) |
|  | High-VHigh Scores | 531 (68.6%) | 127 (16.4%) | 116 (15%)* | 775 (12.1%) |
|  |  |  |  |  |  |
| **Diagnosis of MH** | No Diag/Treat of MH | 4025 (73.1%)* | 866 (15.7%) | 615 (11.2%) | 5506 (86.4%) |
|  | Yes Diag/Treat of MH | 575 (66.2%) | 155 (17.8%) | 139 (16%)* | 869 (13.6%) |
|  |  |  |  |  |  |
| **Occ Group (ANZSCO)** | Managers | 757 (66.6%) | 248 (21.8%)* | 131 (11.5%) | 1136 (17.8%) |
|  | Professionals | 1398 (75.6%)* | 299 (16.2%) | 152 (8.2%) | 1849 (29%) |
|  | Tech/Trades | 847 (67.4%) | 212 (16.9%) | 198 (15.8%)* | 1257 (19.7%) |
|  | Skilled | 1083 (75.4%)* | 178 (12.4%) | 176 (12.2%) | 1437 (22.5%) |
|  | Unskilled | 515 (74.1%) | 83 (11.9%) | 97 (14%) | 695 (10.9%) |
|  |  |  |  |  |  |
| **Household Income** | DK/PNTS | 759 (84.8%)* | 65 (7.3%) | 71 (7.9%) | 895 (14%) |
|  | Low Income | 430 (76.2%)* | 60 (10.6%) | 74 (13.1%) | 564 (8.8%) |
|  | Middle Income | 1244 (73.4%) | 231 (13.6%) | 220 (13%) | 1695 (26.6%) |
|  | High Income | 2167 (67.3%) | 665 (20.6%)* | 390 (12.1%) | 3222 (50.5%) |
| Note: * = Significant category within variable based on Adjusted Standardised Residual value being greater than 1.96.  % for total is based on total category. % for risk groups are for within category.  Abbreviations: YMA = Younger Middle Adults, OMA = Older Middle Adults, Sep = Separated, DNF = Did not Finish, Dip = Diploma, Cert = Certificate, Bach = Bachelors’, SES = Socio-economic status, Dep = Dependent, K10 = Kessler 10 Psychological Scale, Diag = Diagnosis, Treat = Treatment, MH = Mental Health, Occ = Occupation, DK = Don’t know, PNTS = Prefer not to say. | | | | | |

| **Supplementary Table 3**: Initial regression model for drinking in the ‘**exceeded - high risk**’ group of NHMRC 2020 Guidelines (compared to drinking in the ‘**did not exceed**’ group and ‘**exceeded**’ group) based on volume estimates among all midlife men aged 30-59 years from 2019 and 2022/23 NDSHS waves. | | | | |
| --- | --- | --- | --- | --- |
| Variables and Categories |  |  | 95% CI | |
|  | ***p*** | **OR** | Lower Upper | |
| Age Groups (RC: Younger Middle Adults) |  |  |  |  |
| Older Middle Adults | .002* | 1.39 | 1.13 | 1.71 |
| Smoker Status (RC: Former/non-Smoker) |  |  |  |  |
| Currently Smoking | <.001** | 2.52 | 1.95 | 3.25 |
| Used Illicit Drugs (last 12 months) (RC: Non-User) |  |  |  |  |
| Used Last 12 Months | <.001** | 2.48 | 1.97 | 3.11 |
| Marital Status (RC: Single/Never Married) |  |  |  |  |
| Divorced/Widowed/Separated | .55 | 1.12 | 0.77 | 1.64 |
| Married/De Facto | .19 | 0.81 | 0.60 | 1.10 |
| Educational Attainment (RC: DNF High School) |  |  |  |  |
| Completed Year 12 | .06 | 0.67 | 0.44 | 1.01 |
| Diploma/Cert III + | .12 | 0.75 | 0.53 | 1.08 |
| Bachelor’s Degree + | <.001** | 0.46 | 0.30 | 0.69 |
| SEIFA (SES) (RC: 1 – Most Disadvantaged) |  |  |  |  |
| 2 – Slightly Disadvantaged | .09 | 1.35 | 0.95 | 1.90 |
| 3 – Neutral | .23 | 1.25 | 0.86 | 1.81 |
| 4 – Slightly Advantaged | .43 | 1.15 | 0.80 | 1.65 |
| 5 – Most Advantaged | .42 | 1.17 | 0.80 | 1.73 |
| ASGS (Rurality) (RC: Metropolitan) |  |  |  |  |
| Rural/Remote/Regional | .001* | 1.46 | 1.15 | 1.84 |
| Dep Child in Household (RC: No Children) |  |  |  |  |
| 1+ Children | .83 | 0.97 | 0.78 | 1.22 |
| Psychological Distress (K10) (RC: Low-Mod Scores) |  |  |  |  |
| High-Very High Scores | .98 | 0.99 | 0.70 | 1.41 |
| Diagnosis or Treatment MH (RC: No) |  |  |  |  |
| Yes – Diagnosis/Treatment of MH | .35 | 1.17 | 0.84 | 1.63 |
| Occupational Group (RC: Skilled Professions) |  |  |  |  |
| Managers | .47 | 1.14 | 0.80 | 1.63 |
| Professionals | .91 | 1.02 | 0.72 | 1.44 |
| Tech & Trades | .09 | 1.29 | 0.96 | 1.72 |
| Unskilled Professions | .47 | 1.14 | 0.79 | 1.65 |
| Household Income (RC: Mid ($1000-1999 per week)) |  |  |  |  |
| Don’t Know/Prefer not to say | .005* | 0.59 | 0.41 | 0.85 |
| Low ($999 or less per week) | .11 | 0.73 | 0.50 | 1.07 |
| High ($2000 or more per week) | .02* | 1.35 | 1.05 | 1.73 |
| Note: Abbreviations are as follows: RC = Reference Category, DNF = Did not finish, SES = Socio-economic Status, Dep = Dependent, MH = Mental Health  Raw n = 6,439. Relative Weighted n = 6,375  Not Exceeded - High Risk group = 5,615, Exceeded - High Risk group = 824 (RC). | | | | |

To assess the impact of dichotomising the study’s outcome variable (risky drinking as defined by the NHMRC alcohol guidelines), a sensitivity analysis was run using higher thresholds. These higher thresholds were considered by combining answers from the following questions of both the 2019 and 2022-23’s NDSHS: ‘*In the last 12 months, how often did you have an alcoholic drink of any kind? (*E7)*’* and *‘On a day that you have an alcoholic drink, how many standard drinks do you usually have?* (E14)*’*. The answers for each of these questions were then assigned a score defining its midpoint, utilised similarly in a prior study (1). For this analysis, samples were split into three risky drinking groups based on overall scores: **Did not exceed group** (scored 0-519 – defined as those that did not exceed NHMRC guidelines), **Exceeded group** (scored 520-1039 – defined as those that exceeded NHMRC guidelines but did NOT exceed it by double) and **Exceeded - High Risk group** (scored 1040 + - defined as those that exceeded NHMRC guidelines by double or greater).

**Example of midpoint scoring:** A participant responded drinking ‘*3 to 4 days a week’* (E7) and *‘5-6 standard drinks per occasion’* (E14), the E7 Response would receive a score of 3.5 (days a week) multiplied by weeks (52) = 182. This number is then multiplied by the response of E14 (5.5 is the midpoint of 5-6) = a drinking volume score of 1,001, thus placing this respondent in the ‘**Exceeded’** group.

Supplementary table 2 displays the descriptive statistics of all midlife men and their risky drinking groups and the variables of interest. Supplementary table 3 shows the results from the initial logistic regression model, focusing primarily on the **Exceeded - High Risk** **group** when compared to those midlife men that **were not** part of this group. Findings from this regression model show similar findings to the initial regression model from the primary manuscript, where, with the exception of those currently smoking, all existing significant findings were weaker due to lower analytical power. The major changes from this sensitivity analysis were with psychological distress no longer being significant, and OMA being significantly more likely to drink at the ‘**exceeded - high risk**’ group levels when compared to YMA.

Reference

1. Livingston M, Room R. Variations by age and sex in alcohol-related problematic behaviour per drinking volume and heavier drinking occasion. Drug and Alcohol Dependence. 2009;101(3):169-75.
