# Supplementary material for: Risky Drinking in Midlife Men: Insights From Australia's National Drug Strategy Household Survey
Source: Drug Alcohol Rev. 2026 Apr 13;45(4):e70149. doi: 10.1111/dar.70149 (PMC13077182; doi:10.1111/dar.70149)

Summary of respondents excluded due to incomplete/uninterpretable/invalid responses (values based on raw n)

| **Stage** | **Criteria** | **Samples Remaining** | **Samples Excluded on Stage** |
| --- | --- | --- | --- |
| **1** | **Starting Sample (2019 and 2022/23 total)** | 43,678 | 0 |
| **2** | **Responded “Male” and Aged as “Midlife” (30-59 years)** | 9,006 | 34,672 |
| **3** | **Did not respond to Alcohol Guideline Variables** | 8,295 | 711 |
| **4** | **Did not respond to Smoking Variable** | 8,293 | 2 |
| **5** | **Did not respond to Illicit Drug Variable** | 8,029 | 264 |
| **6** | **Did not respond to Marital Status Variable** | 8,004 | 25 |
| **7** | **Did not respond to Education Variable** | 7,663 | 341 |
| **8** | **Did not respond to SES (IRSAD) Variable** | 7,663 | 0 |
| **9** | **Did not respond to Rurality (ASGS) Variable** | 7,663 | 0 |
| **10** | **Did not respond to Dependent Children in Household Variable** | 7,374 | 289 |
| **11** | **Did not respond to Psychological Distress (K10) Variable** | 7,337 | 37 |
| **12** | **Did not respond to Diagnosis and/or Treatment of Mental Health Variable** | 7,172 | 165 |
| **13** | **Did not respond to Occupational Group (ANZSCO) Variable** | 6,508 | 664 |
| **14** | **Did not respond to Household Income Variable** | 6,471 | 37 |
|  | **FINAL SAMPLE** | 6,471 |  |

Stages were determined through including a filter at each stage to show a continuous decrease in samples. For example, beginning at the starting sample, a filter was added to “Select Cases” to only consider “males” and respondents who were aged between and including “30 to 59 years”, filtering out those who did not fit those categories. Each stage would then add another variable to the filter, considering only the samples without missing responses until all variables had been included on the filter.

| \| **Supplementary Table 1**: Demographic information for Younger Middle Adults aged 30-44 years who reported drinking in excess of NHMRC 2020 guideline 1 in 2019/2022/23 NDSHS Waves. Raw *n* = 3,311 (42.3% reported drinking exceeding guideline 1). \| \| \| \| \| \| --- \| --- \| --- \| --- \| --- \| \| Factor \| Demographic \| *n* % \| Risky Drinking Prevalence (95% CI) \| Sig \| \|  \|  \|  \| Weighted = 43.3% \|  \| \|  \|  \|  \|  \|  \| \| Smoker Status \| Former/Non-Smoker \| 1074 (78.8%) \| 40.4% (37.7-43.0) \| χ2 (1, 3296) = 60.27, *p* <.001** \| \| Currently Smoking \| 326 (21.2%) \| 58.9% (53.0-64.5) \| \|  \|  \|  \|  \|  \| \| Illicit Drug use (last 12 months) \| Not using Drugs (last 12 Months) \| 864 (62.5%) \| 35.3% (32.6-38.1) \| χ2 (1, 3296) = 281.31, *p* <.001** \| \| Used Drugs (last 12 Months) \| 536 (37.5%) \| 69.5% (64.8-73.7) \| \|  \|  \|  \|  \|  \| \| Marital Status \| Never Married \| 323 (20.2%) \| 44.8% (39.3-50.4) \| χ2 (2, 3296) = 1.66, *p* =.43 \| \| Divorced/Widowed/Separated \| 88 (4.5%) \| 46.7% (36.2-57.4) \| \| Married/DeFacto \| 989 (75.3%) \| 42.6% (39.9-45.5) \| \|  \|  \|  \|  \|  \| \| Peak Educational Attainment \| Did Not Finish High School \| 124 (8.7%) \| 46.6% (38.9-54.5) \| χ2 (3, 3296) = 68.99, *p* <.001** \| \| Completed Year 12 \| 188 (14.4%) \| 43.6% (36.5-50.9) \| \| Completed Diploma/Cert III + \| 550 (39.6%) \| 52.3% (48.1-56.3) \| \| Completed Bachelors’ Degree + \| 538 (37.3%) \| 36.0% (32.5-39.6) \| \|  \|  \|  \|  \|  \| \| Socio-economic Status (SEIFA-AD) \| 1 – Most Disadvantaged \| 208 (16.5%) \| 40.4% (34.7-46.5) \| χ2 (4, 3296) = 5.84, *p* = .21 \| \| 2 – Slightly Disadvantaged \| 243 (18.4%) \| 42.4% (37.0-47.9) \| \| 3 – Neutral \| 291 (21.5%) \| 46.5% (41.6-51.6) \| \| 4 – Slightly Advantaged \| 319 (22.6%) \| 44.5% (39.0-50.1) \| \| 5 – Most Advantaged \| 339 (21.0%) \| 42.0% (36.6-47.4) \| \|  \|  \|  \|  \|  \| \| Rurality (ASGS) \| Metropolitan \| 967 (71.4%) \| 40.3% (37.5-43.2) \| χ2 (1, 3296) = 38.31, *p* <.001** \| \| Rural/Regional/Remote \| 433 (28.6%) \| 52.9% (48.3-57.5) \| \|  \|  \|  \|  \|  \| \| Dependent Children in HH \| No Dependent Children in HH \| 570 (37.0%) \| 42.3% (38.5-46.1) \| χ2 (1, 3296) = .82, *p* =.36 \| \| 1+ Dependent Children in HH \| 830 (63.0%) \| 43.9% (40.7-47.0) \| \|  \|  \|  \|  \|  \| \| Psychological Distress (K10) \| Low-Med Psych Dist K10 Score \| 1168 (82.6%) \| 41.1% (38.5-43.7) \| χ2 (1, 3296) = 41.91, *p* <.001** \| \| High-VHigh Psych Dist K10 Score \| 232 (17.4%) \| 57.7% (51.1-64.1) \| \|  \|  \|  \|  \|  \| \| Diagnosis and Treatment of MH \| No Diagnosis/Treatment of MH \| 1181 (84.6%) \| 42.5% (39.9-45.2) \| χ2 (1, 3296) = 4.72, *p* =.03* \| \| Yes Diagnosis/Treatment of MH \| 219 (15.4%) \| 47.9% (41.0-54.9) \| \|  \|  \|  \|  \|  \| \| Occupational Group (ANZSCO) \| Manager Occupation \| 279 (19.4%) \| 51.1% (45.2-57.0) \| χ2 (4, 3296) = 55.60, *p* <.001** \| \| Professional Occupation \| 397 (27.6%) \| 38.5% (34.3-42.9) \| \| Tech & Trade Occupation \| 329 (24.2%) \| 52.1% (47.0-57.3) \| \| Skilled Occupation \| 255 (17.8%) \| 37.3% (32.2-42.8) \| \| Unskilled Occupation \| 140 (11.1%) \| 39.9% (33.4-46.8) \| \|  \|  \|  \|  \|  \| \| Household Income \| Don’t Know/Prefer not to say HHI \| 120 (8.4%) \| 27.7% (22.5-33.6) \| χ2 (3, 3296) = 89.35, *p* <.001** \| \| Low - $999 or less per week HHI \| 85 (6.0%) \| 33.0% (24.4-42.6) \| \| Mid - $1000-$1999 per week HHI \| 387 (25.9%) \| 40.6% (36.1-45.3) \| \| High - $2000 or more per week HHI \| 808 (59.7%) \| 50.2% (46.8-53.6) \| \| Key: *= category is significant as p <.05, ** = category is highly significant as p <.001  Abbreviations are as follows: ASGS = Rurality, HH = Household, Psych Dist K10 = Psychological Distress Kessler 10, MH = Mental Health, HHI = Household Income  Raw n for “Yes” Response = 1,400, weighted n = 1,426. Raw n for “No” Response = 1,911, weighted n = 1,870  Note: Raw samples and relative weighted percentage and significance values were reported. \| \| \| \| \| |
| --- | --- | --- | --- | --- | --- | --- | --- | --- | --- | --- | --- | --- | --- | --- | --- | --- | --- | --- | --- | --- | --- | --- | --- | --- | --- | --- | --- | --- | --- | --- | --- | --- | --- | --- | --- | --- | --- | --- | --- | --- | --- | --- | --- | --- | --- | --- | --- | --- | --- | --- | --- | --- | --- | --- | --- | --- | --- | --- | --- | --- | --- | --- | --- | --- | --- | --- | --- | --- | --- | --- | --- | --- | --- | --- | --- | --- | --- | --- | --- | --- | --- | --- | --- | --- | --- | --- | --- | --- | --- | --- | --- | --- | --- | --- | --- | --- | --- | --- | --- | --- | --- | --- | --- | --- | --- | --- | --- | --- | --- | --- | --- | --- | --- | --- | --- | --- | --- | --- | --- | --- | --- | --- | --- | --- | --- | --- | --- | --- | --- | --- | --- | --- | --- | --- | --- | --- | --- | --- | --- | --- | --- | --- | --- | --- | --- | --- | --- | --- | --- | --- | --- | --- | --- | --- | --- | --- | --- | --- | --- | --- | --- | --- | --- | --- | --- | --- | --- | --- | --- | --- | --- | --- | --- | --- | --- | --- | --- | --- | --- | --- | --- | --- | --- | --- | --- | --- | --- | --- | --- | --- | --- | --- | --- | --- | --- | --- |

| **Supplementary Table 2**: Demographic information for Older Middle Adults aged 45-59 years who reported drinking in excess of NHMRC 2020 guideline 1 in 2019/2022/23 NDSHS Waves. Raw *n* = 3,160 (44.3% reported drinking exceeding guideline 1). | | | | |
| --- | --- | --- | --- | --- |
| Factor | Demographic | *n* % | Risky Drinking Prevalence (95% CI) | Sig |
|  |  |  | Weighted = 42.6% |  |
|  |  |  |  |  |
| Smoker Status | Former/Non-Smoker | 1083 (78.2%) | 39.6% (36.9-42.2) | χ2 (1, 3112) = 62.95, *p* <.001** |
|  | Currently Smoking | 317 (21.8%) | 58.9% (52.9-64.7) |  |
|  |  |  |  |  |
| Illicit Drug use (last 12 months) | Not using Drugs (last 12 Months) | 1004 (72.9%) | 37.3% (34.8-39.9) | χ2 (1, 3112) = 175.62, *p* <.001** |
|  | Used Drugs (last 12 Months) | 396 (27.1%) | 68.8% (62.9-74.0) |  |
|  |  |  |  |  |
| Marital Status | Never Married | 174 (7.5%) | 34.3% (27.4-42.0) | χ2 (2, 3112) = 13.63, *p* =.001* |
|  | Divorced/Widowed/Separated | 224 (14.1%) | 48.4% (40.6-56.2) |  |
|  | Married/DeFacto | 1002 (78.4%) | 42.7% (39.9-45.4) |  |
|  |  |  |  |  |
| Peak Educational Attainment | Did Not Finish High School | 230 (15.5%) | 46.6% (40.7-52.6) | χ2 (3, 3112) = 26.92, *p* <.001** |
|  | Completed Year 12 | 181 (12.3%) | 40.6% (33.8-47.8) |  |
|  | Completed Diploma/Cert III + | 567 (40.6%) | 47.2% (43.1-51.3) |  |
|  | Completed Bachelors’ Degree + | 422 (31.6%) | 37.1% (33.1-41.3) |  |
|  |  |  |  |  |
| Socio-economic Status (SEIFA-AD) | 1 – Most Disadvantaged | 183 (11.6%) | 35.3% (29.5-41.6) | χ2 (4, 3112) = 28.54, *p* <.001** |
|  | 2 – Slightly Disadvantaged | 266 (19.7%) | 48.0% (42.3-53.7) |  |
|  | 3 – Neutral | 287 (23.4%) | 43.3% (37.9-48.9) |  |
|  | 4 – Slightly Advantaged | 313 (21.3%) | 38.0% (33.3-43.0) |  |
|  | 5 – Most Advantaged | 351 (23.9%) | 47.2% (42.0-52.5) |  |
|  |  |  |  |  |
| Rurality (ASGS) | Metropolitan | 926 (68.0%) | 39.2% (36.3-42.1) | χ2 (1, 3112) = 41.47, *p* <.001** |
|  | Rural/Regional/Remote | 474 (32.0%) | 52.2% (47.9-56.5) |  |
|  |  |  |  |  |
| Dependent Children in HH | No Dependent Children in HH | 792 (48.0%) | 44.4% (40.9-48.0) | χ2 (1, 3112) = 3.53, *p* =.06 |
|  | 1+ Dependent Children in HH | 608 (52.0%) | 41.1% (37.7-44.5) |  |
|  |  |  |  |  |
| Psychological Distress (K10) | Low-Med Psych Dist K10 Score | 1232 (89.0%) | 42.7% (40.1-45.4) | χ2 (1, 3112) = .17, *p* = .68 |
|  | High-VHigh Psych Dist K10 Score | 168 (11.0%) | 41.6% (34.5-49.2) |  |
|  |  |  |  |  |
| Diagnosis and Treatment of MH | No Diagnosis/Treatment of MH | 1198 (86.3%) | 42.5% (39.9-45.2) | χ2 (1, 3112) = .03, *p* =.86 |
|  | Yes Diagnosis/Treatment of MH | 202 (13.7%) | 43.0% (36.7-49.5) |  |
|  |  |  |  |  |
| Occupational Group (ANZSCO) | Manager Occupation | 314 (21.1%) | 46.9% (41.4-52.6) | χ2 (4, 3112) = 23.47, *p* <.001** |
|  | Professional Occupation | 345 (38.7%) | 38.6% (34.2-43.3) |  |
|  | Tech & Trade Occupation | 284 (22.4%) | 48.6% (42.9-54.3) |  |
|  | Skilled Occupation | 311 (22.2%) | 38.6% (33.8-43.7) |  |
|  | Unskilled Occupation | 146 (9.8%) | 42.9% (35.6-50.6) |  |
|  |  |  |  |  |
| Household Income | Don’t Know/Prefer not to say HHI | 147 (9.9%) | 27.8% (22.7-33.6) | χ2 (3, 3112) = 75.20, *p* <.001** |
|  | Low - $999 or less per week HHI | 130 (7.9%) | 34.3% (27.4-41.8) |  |
|  | Mid - $1000-$1999 per week HHI | 364 (25.4%) | 42.5% (37.7-47.4) |  |
|  | High - $2000 or more per week HHI | 759 (56.8%) | 48.8% (45.3-52.4) |  |
| Key: *= category is significant as p <.05, ** = category is highly significant as p <.001  Abbreviations are as follows: ASGS = Rurality, HH = Household, Psych Dist K10 = Psychological Distress Kessler 10, MH = Mental Health, HHI = Household Income  Raw n for “Yes” Response = 1,400, weighted n = 1,326. Raw n for “No” Response = 1,760, weighted n = 1,786  Note: Raw samples and relative weighted percentage and significance values were reported. | | | | |

Comparing YMA and OMA separately, five differences within the findings were uncovered. First, YMA males were significantly more prevalent with risky drinking if they reported higher psychological distress scores (57.7% [51.1-64.1]) when compared to OMA. Additionally, YMA males were also significantly more prevalent to risky drinking if they had reported being diagnosed or treated for any mental health condition (47.9% [41.0-54.9]) when compared to OMA. In contrast, the findings for OMA males uncovered significantly higher prevalence in risky drinking if they reported being divorced, widowed or separated (48.4% [40.6-56.2]) when compared to YMA. Additionally, OMA males who reported never marrying were found to be a of significantly lower prevalence of risky drinking (34.3% [27.4-42.0]). Finally, OMA males were significantly more prevalent in risky drinking if they resided in quintiles 2 SES (slightly disadvantaged) (48.0% [42.3-53.7]) and 5 SES (most advantaged) (47.2% [42.0-52.5]) while no significant difference in prevalence was identified among YMA for this demographic. Additionally, OMA residing in quintiles 1 SES (most disadvantaged) (35.3% [29.5-41.6]) and 4 SES (slightly advantaged) (38.0% [33.3-43.0]) had significantly lower prevalence to drinking exceeding guidelines.

| **Figure 1**: Interactions between Age Groups and Smoking Status | | | | |
| --- | --- | --- | --- | --- |
| Variables and Categories |  | 95% CI | |  |
|  | **OR** | Lower | Upper | ***p*** |
| Smoker Status (RC: Former/non-Smoker) |  |  |  |  |
| Currently Smoking | 2.11 | 1.63 | 2.74 | <.001** |
| Age Group (RC: YMA) |  |  |  |  |
| OMA | 0.97 | 0.83 | 1.13 | .67 |
| Age Groups x Smoker Status (RC: YMA x Former/Non) |  |  |  |  |
| OMA x Currently Smoking | 1.04 | 0.71 | 1.51 | .85 |

**Supplementary Figure 1.1:** Interaction between Age Groups and Smoker Status predicting drinking in excess of the Australian National Health and Medical Research Council Guidelines (NHMRC, 2020)


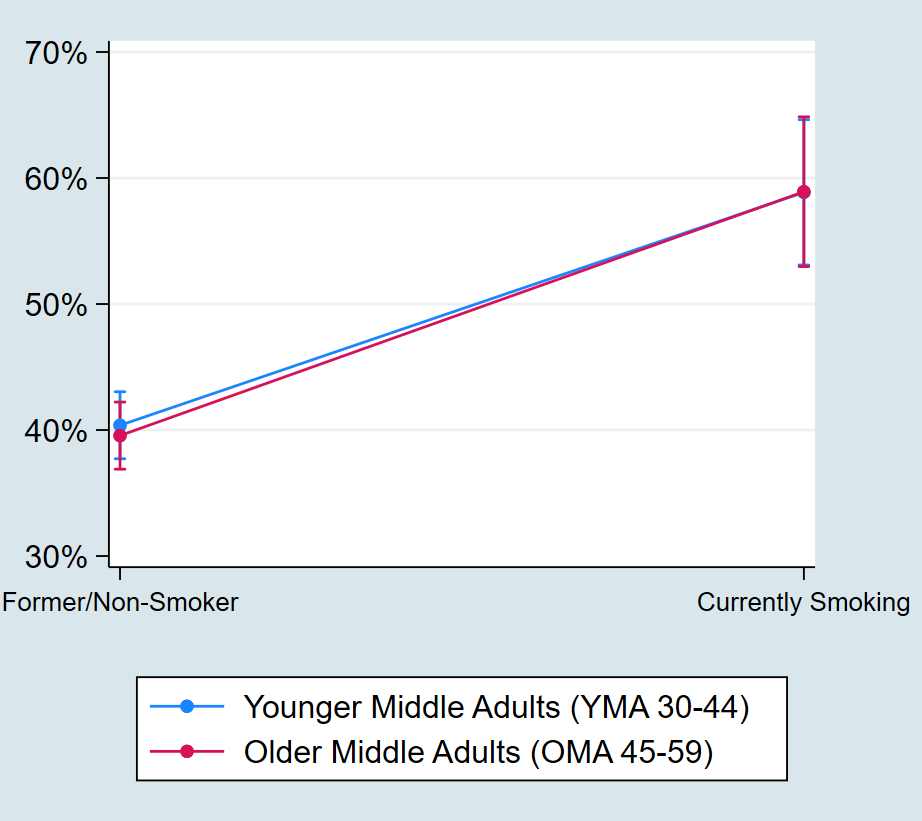


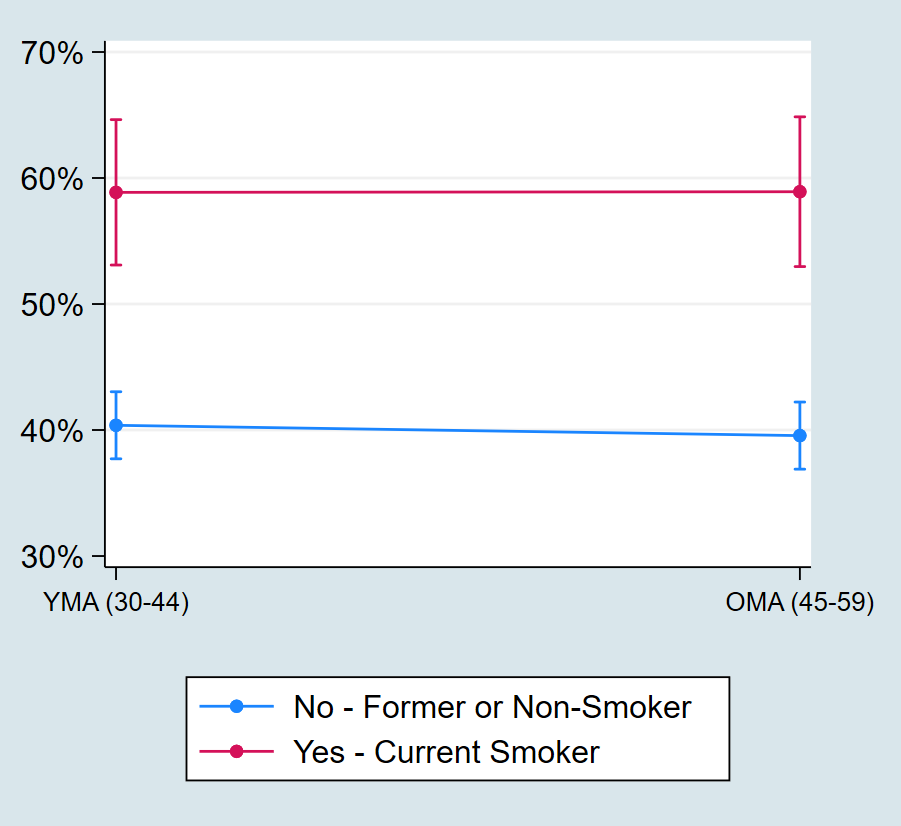


**Supplementary Figure 1.2:** Interaction between Smoker Status and Age Groups predicting drinking in excess of the Australian National Health and Medical Research Council Guidelines (NHMRC, 2020)

| **Figure 2**: Interactions between Age Groups and Used Illicit/non-medicinal use of drugs in last 12 Months | | | | |
| --- | --- | --- | --- | --- |
| Variables and Categories |  | 95% CI | |  |
|  | **OR** | Lower | Upper | ***p*** |
| Illicit Drug User (last 12 months) (RC: Did not use) |  |  |  |  |
| Used Illicit Drugs (last 12 months) | 4.17 | 3.27 | 5.31 | <.001** |
| Age Group (RC: YMA) |  |  |  |  |
| OMA | 1.09 | 0.93 | 1.28 | .28 |
| Age Groups x Illicit Drug User (RC: YMA x Did not use) |  |  |  |  |
| OMA x Used Illicit Drugs (last 12 months) | 0.89 | 0.61 | 1.28 | .52 |


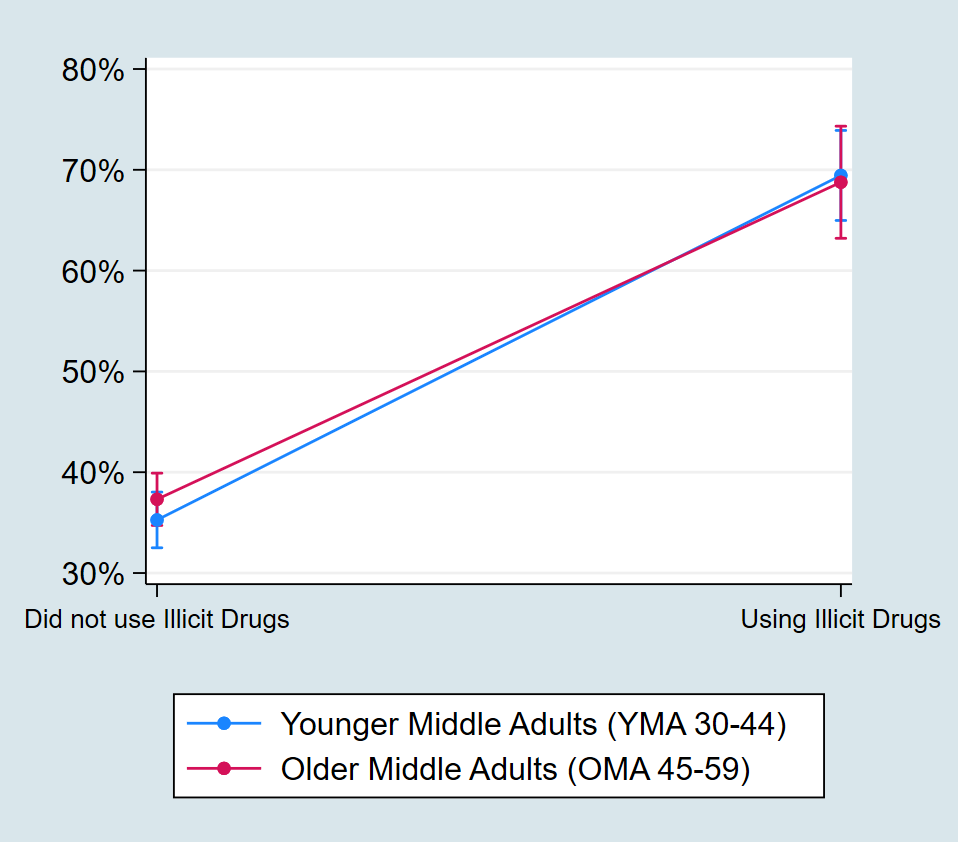


**Supplementary Figure 2.1:** Interaction between Age Groups and Illicit and/or non-medicinal drug use in the last 12 months predicting drinking in excess of the Australian National Health and Medical Research Council Guidelines (NHMRC, 2020)


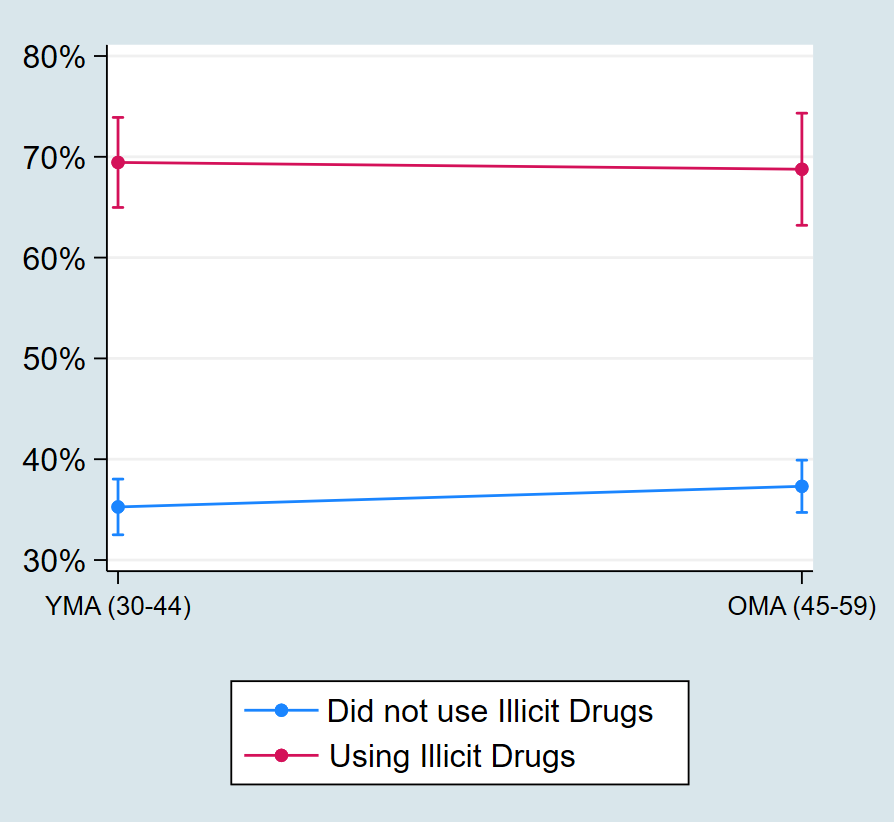


**Supplementary Figure 2.2:** Interaction between Illicit and/or non-medicinal use in the last 12 months and Age Groups predicting drinking in excess of the Australian National Health and Medical Research Council Guidelines (NHMRC, 2020)

| **Figure 3**: Interactions between Age Groups and Marital Status | | | | |
| --- | --- | --- | --- | --- |
| Variables and Categories |  | 95% CI | |  |
|  | **OR** | Lower | Upper | ***p*** |
| Marital Status (RC: Never Married) |  |  |  |  |
| Divorced/Widowed/Separated | 1.08 | 0.66 | 1.76 | .77 |
| Married/DeFacto | 0.91 | 0.71 | 1.18 | .49 |
| Age Group (RC: YMA) |  |  |  |  |
| OMA | 0.64 | 0.43 | 0.96 | .03* |
| Age Groups x Marital Status (RC: YMA x Never Married) |  |  |  |  |
| OMA x Divorced/Widowed/Separated | 1.67 | 0.86 | 3.25 | .13 |
| OMA x Married/DeFacto | 1.55 | 1.01 | 2.39 | .04* |


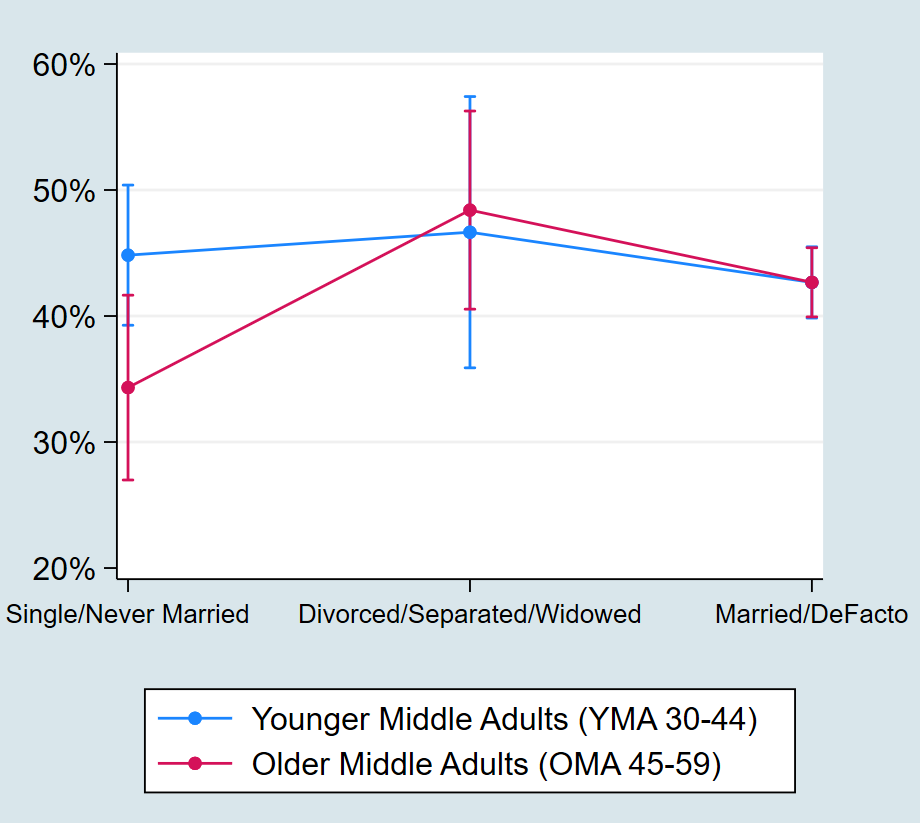


**Supplementary Figure 3.1:** Interaction between Age Groups and Marital Status predicting drinking in excess of the Australian National Health and Medical Research Council Guidelines (NHMRC, 2020)


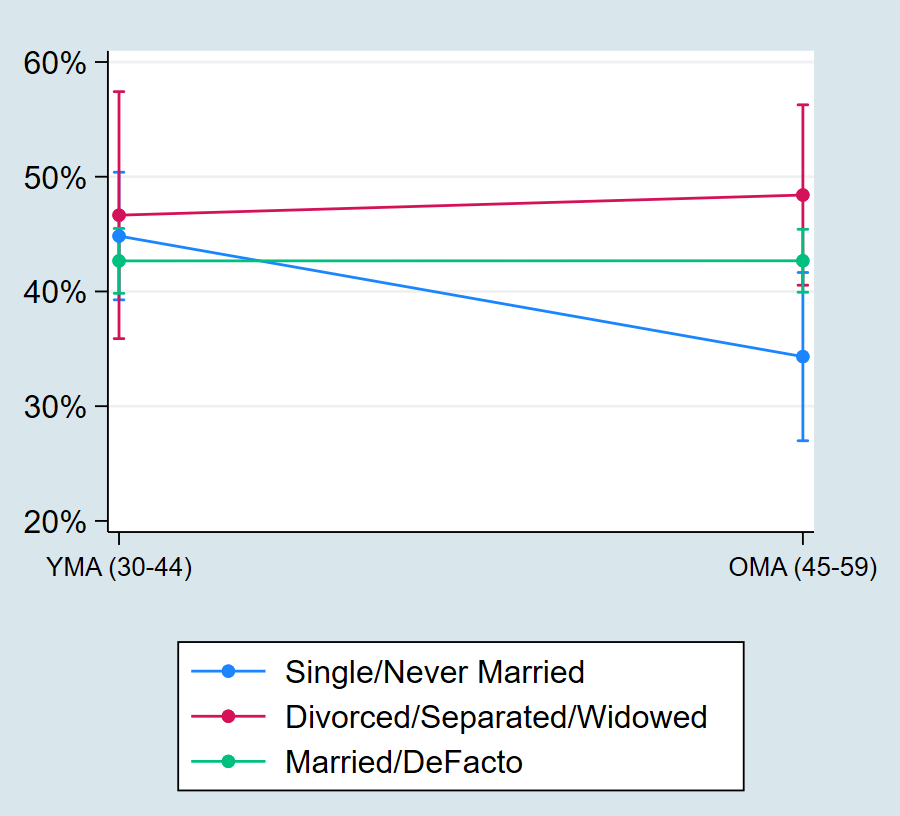


**Supplementary Figure 3.2:** Interaction between Marital Status and Age Groups predicting drinking in excess of the Australian National Health and Medical Research Council Guidelines (NHMRC, 2020)

| **Figure 4**: Interactions between Age Groups and Peak Educational Attainment | | | | |
| --- | --- | --- | --- | --- |
| Variables and Categories |  | 95% CI | |  |
|  | **OR** | Lower | Upper | ***p*** |
| Peak Educational Attainment (RC: DNF Year 12) |  |  |  |  |
| Completed Year 12 | 0.88 | 0.57 | 1.36 | .58 |
| Completed Diploma/Cert III + | 1.25 | 0.88 | 1.79 | .21 |
| Completed Bachelors’ Degree + | 0.64 | 0.45 | 0.91 | .01* |
| Age Group (RC: YMA) |  |  |  |  |
| OMA | 1.00 | 0.68 | 1.47 | .99 |
| Age Groups x Peak Educational Attainment (RC: YMA x DNF Year 12) |  |  |  |  |
| OMA x Completed Year 12 | 0.88 | 0.50 | 1.56 | .67 |
| OMA x Completed Diploma/Cert III + | 0.81 | 0.52 | 1.28 | .38 |
| OMA x Completed Bachelors’ Degree + | 1.05 | 0.67 | 1.64 | .83 |

**Supplementary Figure 4.1:** Interaction between Age Groups and Peak Educational Attainment predicting drinking in excess of the Australian National Health and Medical Research Council Guidelines (NHMRC, 2020)


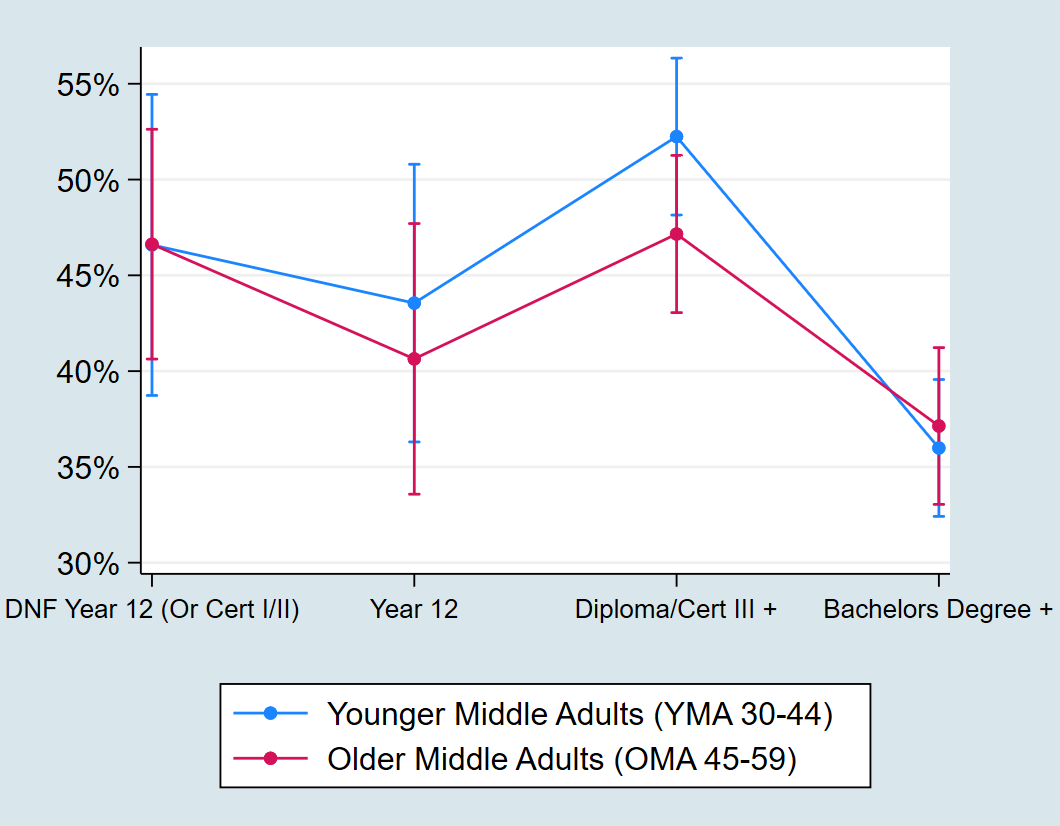


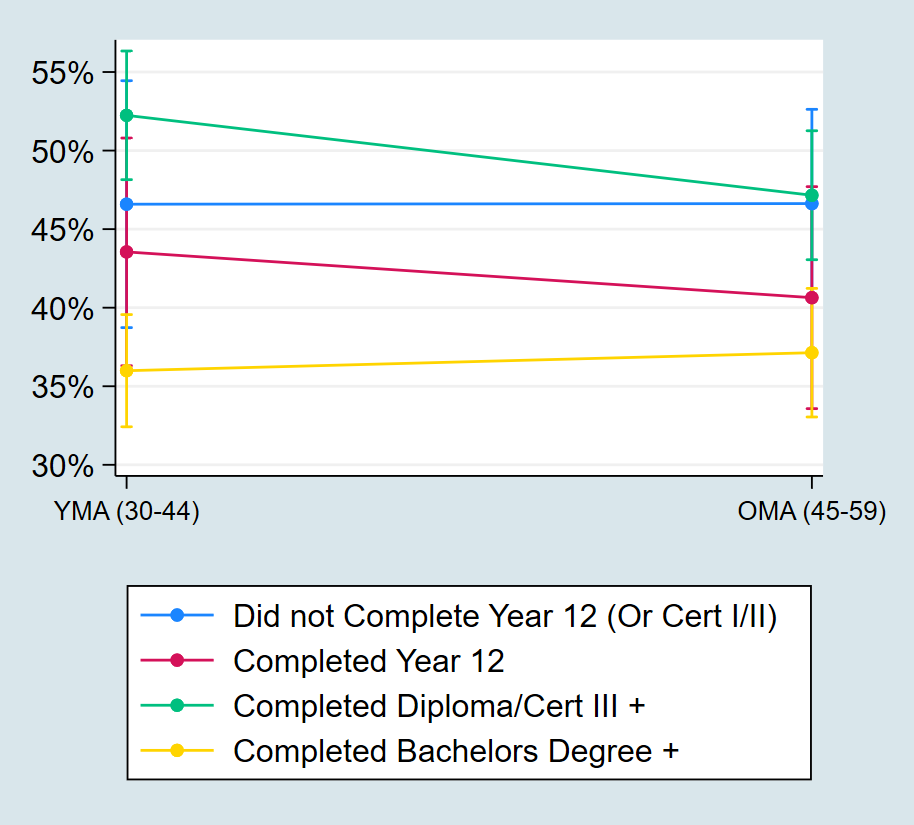


**Supplementary Figure 4.2:** Interaction between Peak Educational Attainment and Age Groups predicting drinking in excess of the Australian National Health and Medical Research Council Guidelines (NHMRC, 2020)

| **Figure 5**: Interactions between Age Groups and Socio-economic Status (Based on SEIFA-AD 2016) | | | | |
| --- | --- | --- | --- | --- |
| Variables and Categories |  | 95% CI | |  |
|  | **OR** | Lower | Upper | ***p*** |
| SEIFA (SES) (RC: Low (1-2)) |  |  |  |  |
| High (3-5) | 1.12 | 0.91 | 1.38 | .28 |
| Age Group (RC: YMA) |  |  |  |  |
| OMA | 1.04 | 0.82 | 1.31 | .75 |
| Age Groups x SEIFA (RC: YMA x Low (1-2)) |  |  |  |  |
| OMA x High (3-5) | 0.90 | 0.67 | 1.21 | .50 |


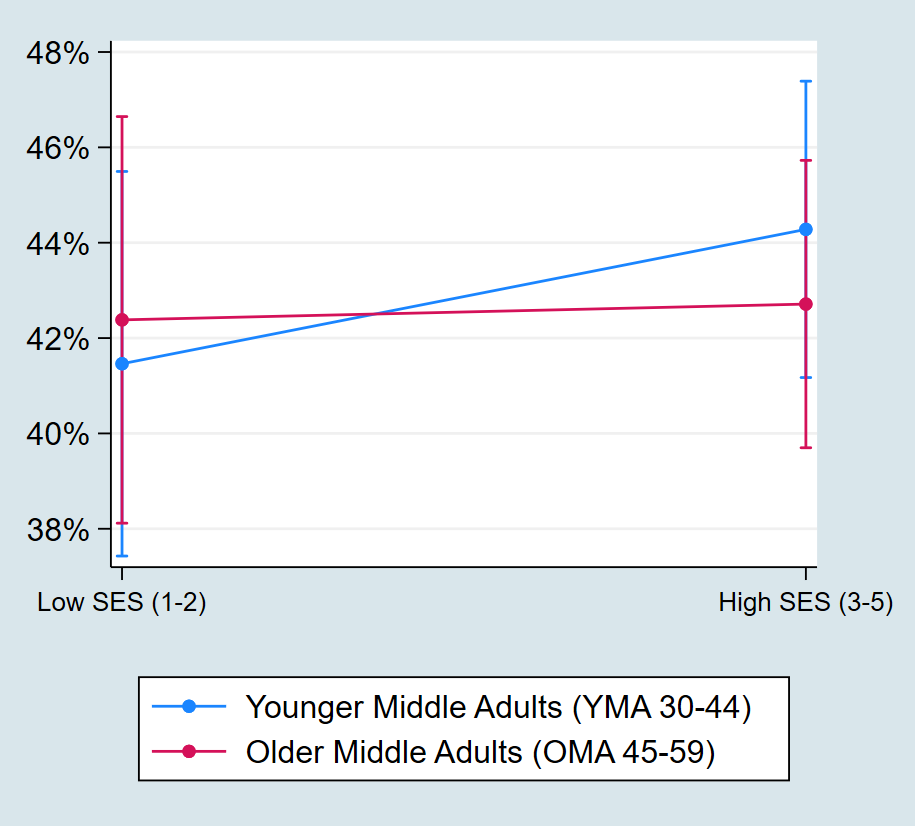


**Supplementary Figure 9:** Interaction between Age Groups and Socio-Economic Status (SEIFA) (ABS, 2023) predicting drinking in excess of the Australian National Health and Medical Research Council Guidelines (NHMRC, 2020)

**Supplementary Figure 5.1:** Interaction between Age Groups and Socio-Economic Status (SEIFA-AD) (ABS, 2023) predicting drinking in excess of the Australian National Health and Medical Research Council Guidelines (NHMRC, 2020)


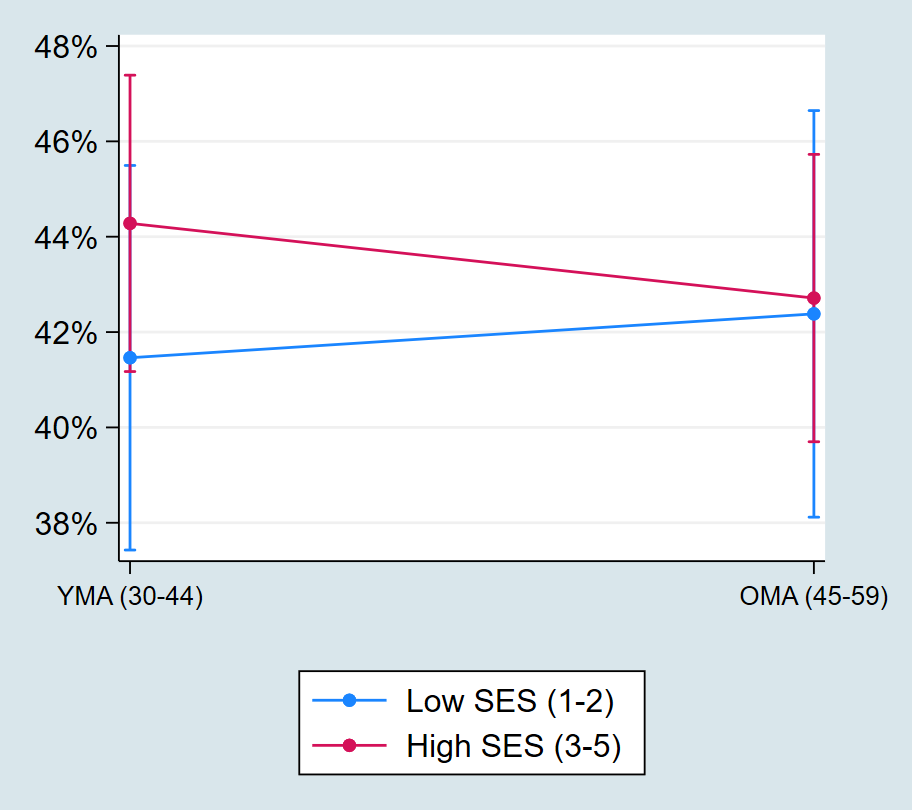


**Supplementary Figure 5.2:** Interaction between Socio-Economic Status (SEIFA-AD) (ABS, 2023) and Age Groups predicting drinking in excess of the Australian National Health and Medical Research Council Guidelines (NHMRC, 2020)

| **Figure 6**: Interactions between Age Groups and Rurality (based on ASGS) | | | | |
| --- | --- | --- | --- | --- |
| Variables and Categories |  | 95% CI | |  |
|  | **OR** | Lower | Upper | ***p*** |
| ASGS (Rurality) (RC: Metropolitan) |  |  |  |  |
| Rural/Remote/Regional | 1.66 | 1.34 | 2.07 | <.001** |
| Age Group (RC: YMA) |  |  |  |  |
| OMA | .95 | 0.81 | 1.13 | .60 |
| Age Groups x ASGS (Rurality) (RC: YMA x Metropolitan) |  |  |  |  |
| OMA x Rural/Remote/Regional | 1.02 | 0.75 | 1.37 | .91 |


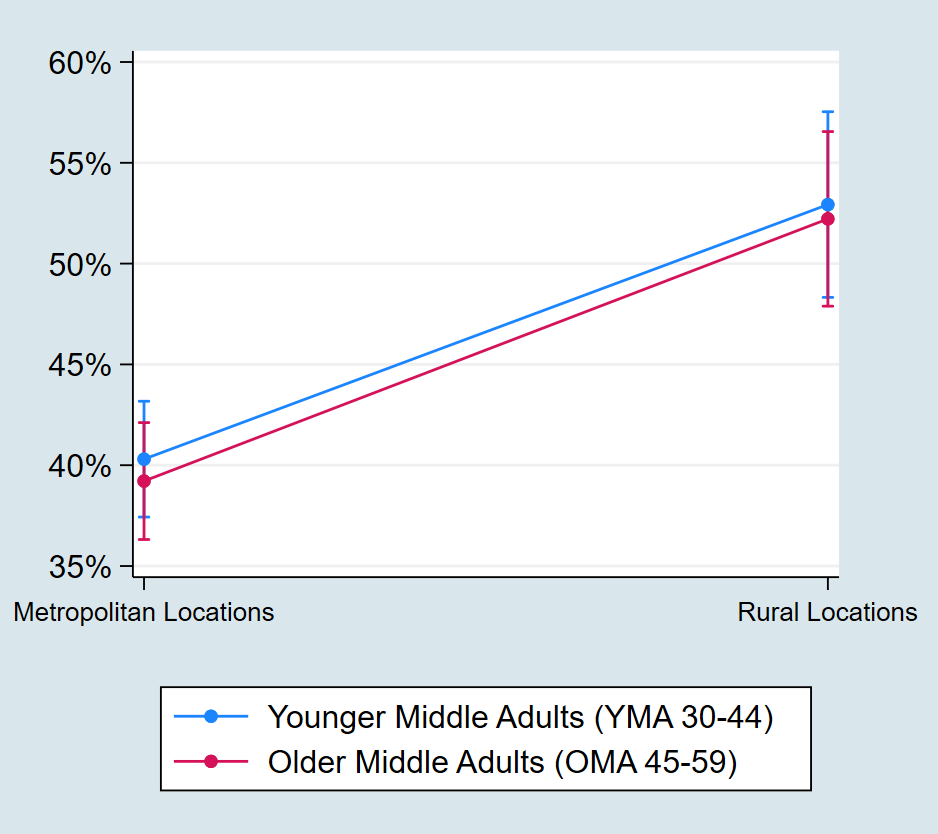


**Supplementary Figure 6.1:** Interaction between Age Groups and Rurality based on the Australian Statistical Demographic Standard (ASGS) (ABS, 2023) predicting drinking in excess of the Australian National Health and Medical Research Council Guidelines (NHMRC, 2020)


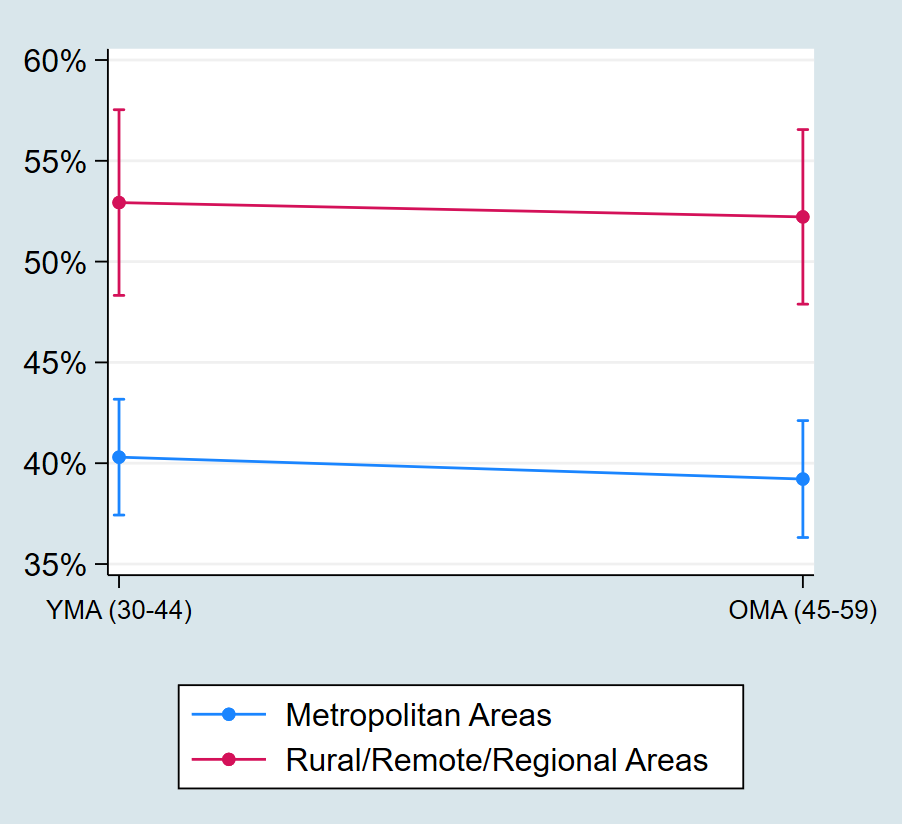


**Supplementary Figure 6.2:** Interaction between Rurality based on the Australian Statistical Demographic Standard (ASGS) (ABS, 2023) and Age Groups predicting drinking in excess of the Australian National Health and Medical Research Council Guidelines (NHMRC, 2020)

| **Figure 7**: Interactions between Age Groups and Dependent Children Living in Household (HH) | | | | |
| --- | --- | --- | --- | --- |
| Variables and Categories |  | 95% CI | |  |
|  | **OR** | Lower | Upper | ***p*** |
| Dependent Children living in HH (Ref: No Children) |  |  |  |  |
| 1+ Children | 1.07 | 0.87 | 1.30 | .52 |
| Age Group (RC: YMA) |  |  |  |  |
| OMA | 1.09 | 0.88 | 1.35 | .42 |
| Age Groups x Dep Child in HH (RC: YMA x No Children) |  |  |  |  |
| OMA x 1+ Children | 0.82 | 0.61 | 1.09 | .16 |


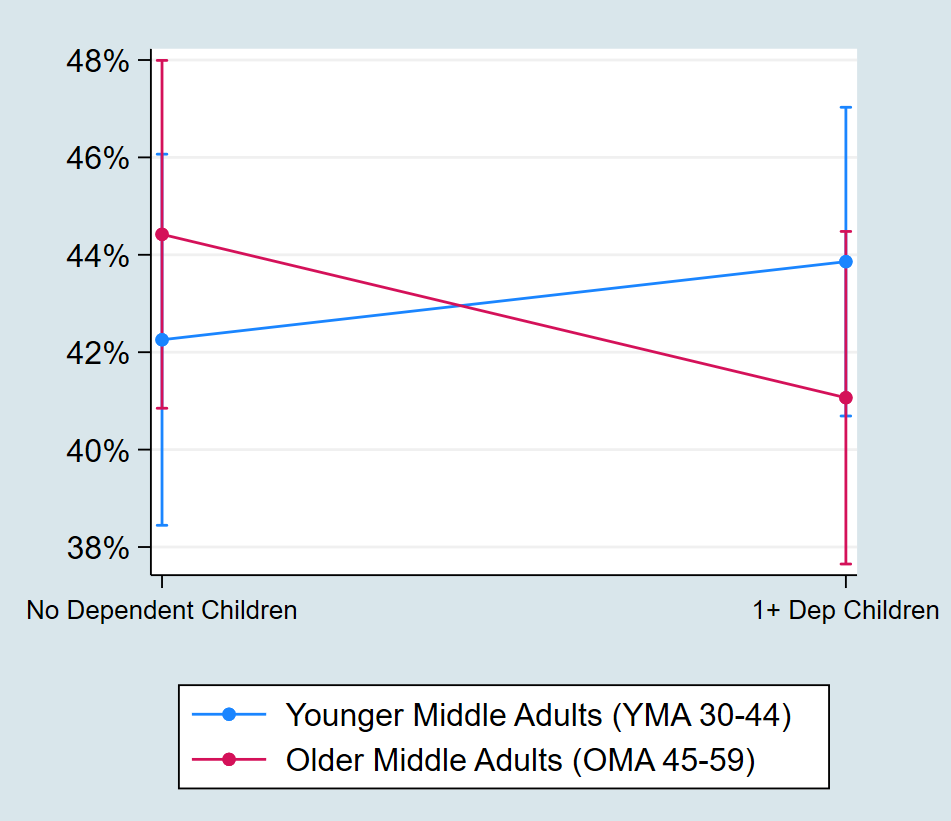


**Supplementary Figure 7.1:** Interaction between Age Groups and Dependent Children Living in the Household predicting drinking in excess of the Australian National Health and Medical Research Council Guidelines (NHMRC, 2020)


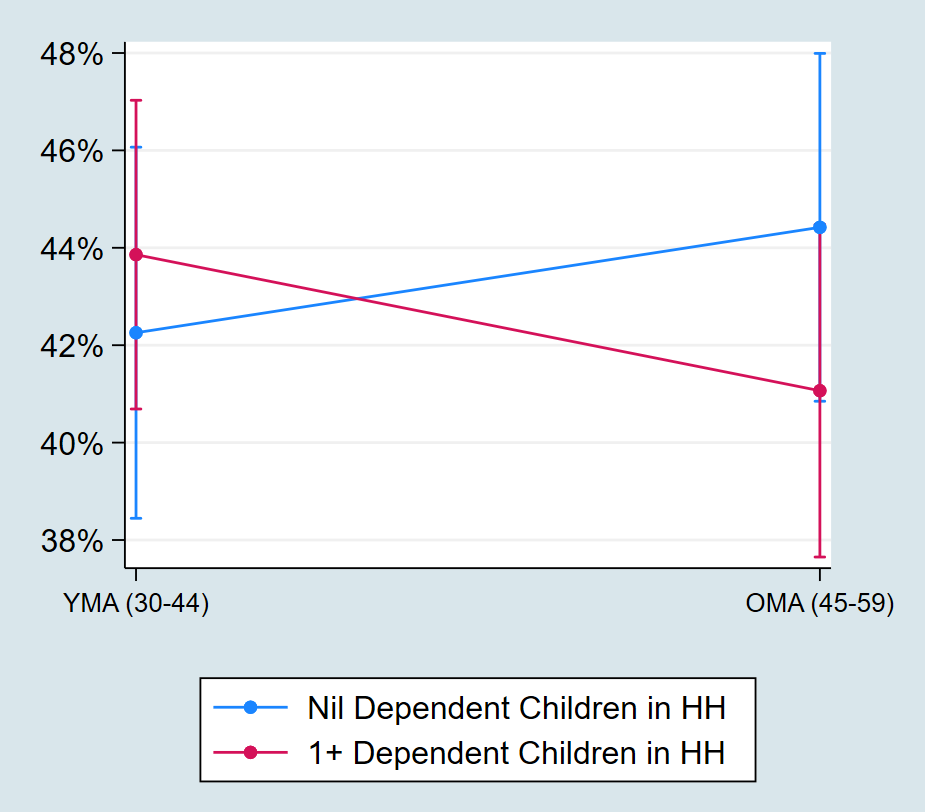


**Supplementary Figure 7.2:** Interaction between Dependent Children Living in the Household and Age Groups predicting drinking in excess of the Australian National Health and Medical Research Council Guidelines (NHMRC, 2020)

| **Figure 8**: Interactions between Age Groups and Psychological Distress scores based on Kessler 10 Scale | | | | |
| --- | --- | --- | --- | --- |
| Variables and Categories |  | 95% CI | |  |
|  | **OR** | Lower | Upper | ***p*** |
| Psychological Distress Scores (K10) (RC: Low-Mod) |  |  |  |  |
| High-Very High | 1.96 | 1.47 | 2.61 | <.001** |
| Age Group (RC: YMA) |  |  |  |  |
| OMA | 1.07 | 0.92 | 1.24 | .38 |
| Age Groups x Psychological Distress Scores (K10) (RC: YMA x Low-Mod) |  |  |  |  |
| OMA x High-Very High | 0.49 | 0.32 | 0.75 | .001* |


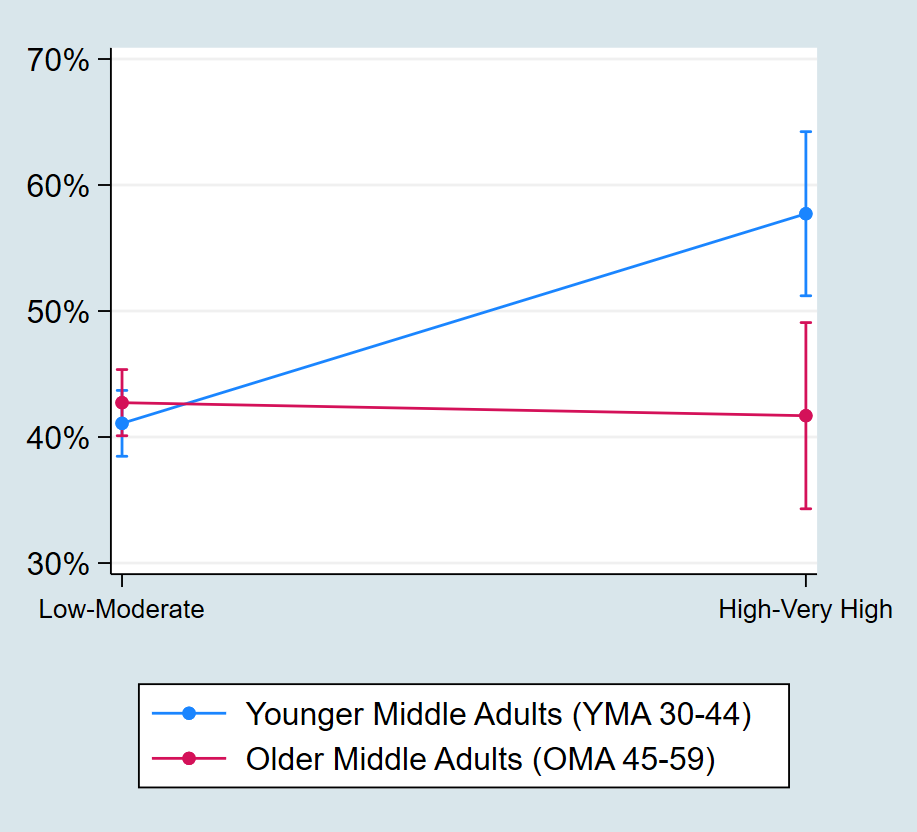


**Supplementary Figure 8.1:** Interaction between Age Groups and Psychological Distress Scores from the Kessler-10 Scale (Kessler et al. 2003) predicting drinking in excess of the Australian National Health and Medical Research Council Guidelines (NHMRC, 2020)


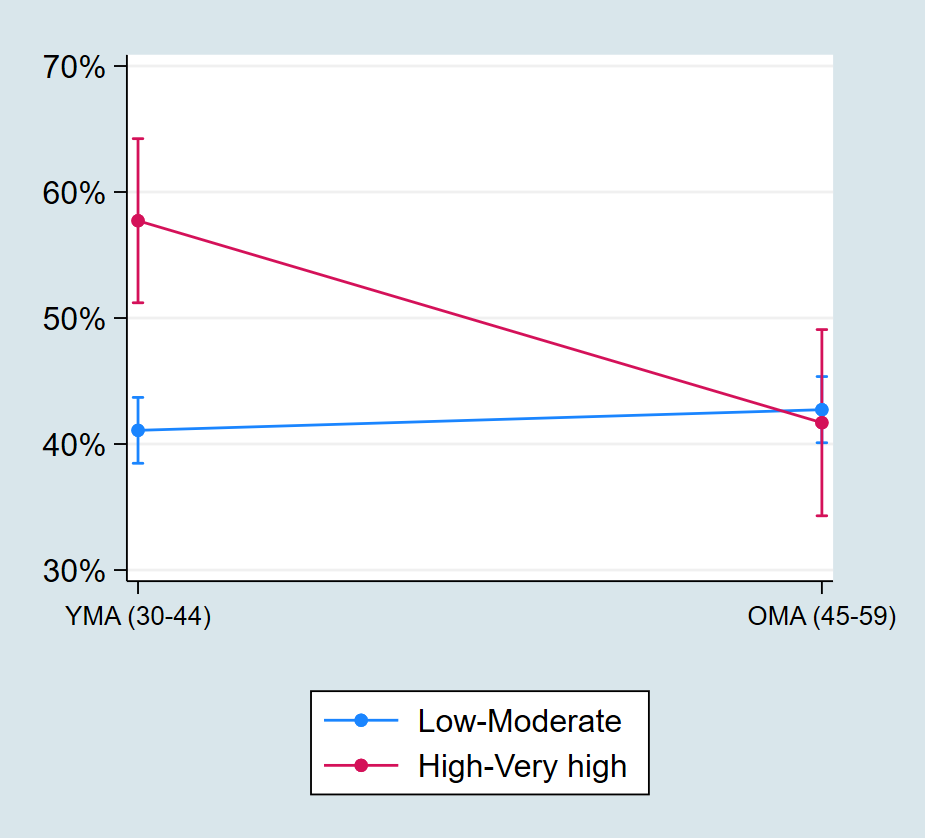


**Supplementary Figure 8.2:** Interaction between Psychological Distress Scores from the Kessler-10 Scale (Kessler et al. 2003) and Age Groups predicting drinking in excess of the Australian National Health and Medical Research Council Guidelines (NHMRC, 2020)

| **Figure 9**: Interactions between Age Groups and Diagnosis/Treatment of Mental Health Condition | | | | |
| --- | --- | --- | --- | --- |
| Variables and Categories |  | 95% CI | |  |
|  | **OR** | Lower | Upper | ***p*** |
| Diagnosed/Treated for MH (RC: No) |  |  |  |  |
| Yes | 1.24 | 0.92 | 1.68 | .16 |
| Age Group (RC: YMA) |  |  |  |  |
| OMA | 1.00 | 0.86 | 1.17 | .98 |
| Age Groups x Diagnosed/Treated for MH (RC: YMA x No) |  |  |  |  |
| OMA x Yes | 0.82 | 0.54 | 1.24 | .34 |


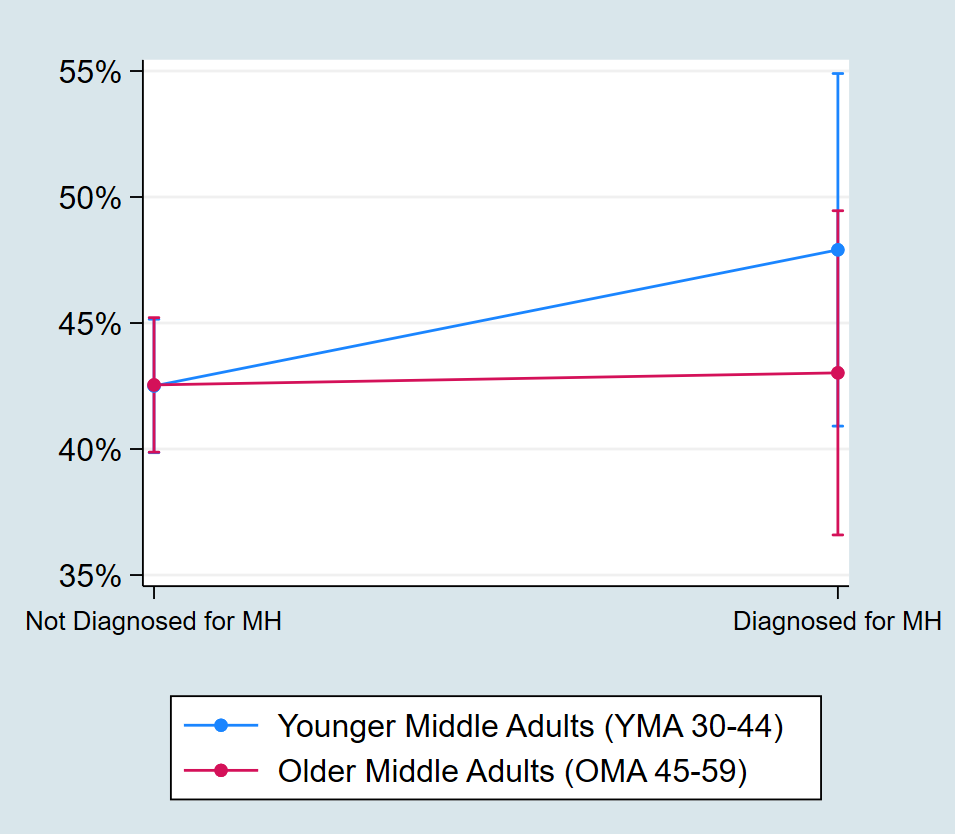


**Supplementary Figure 9.1:** Interaction between Age Groups and Having a Diagnosis and/or Treated for any Mental Health Condition predicting drinking in excess of the Australian National Health and Medical Research Council Guidelines (NHMRC, 2020)


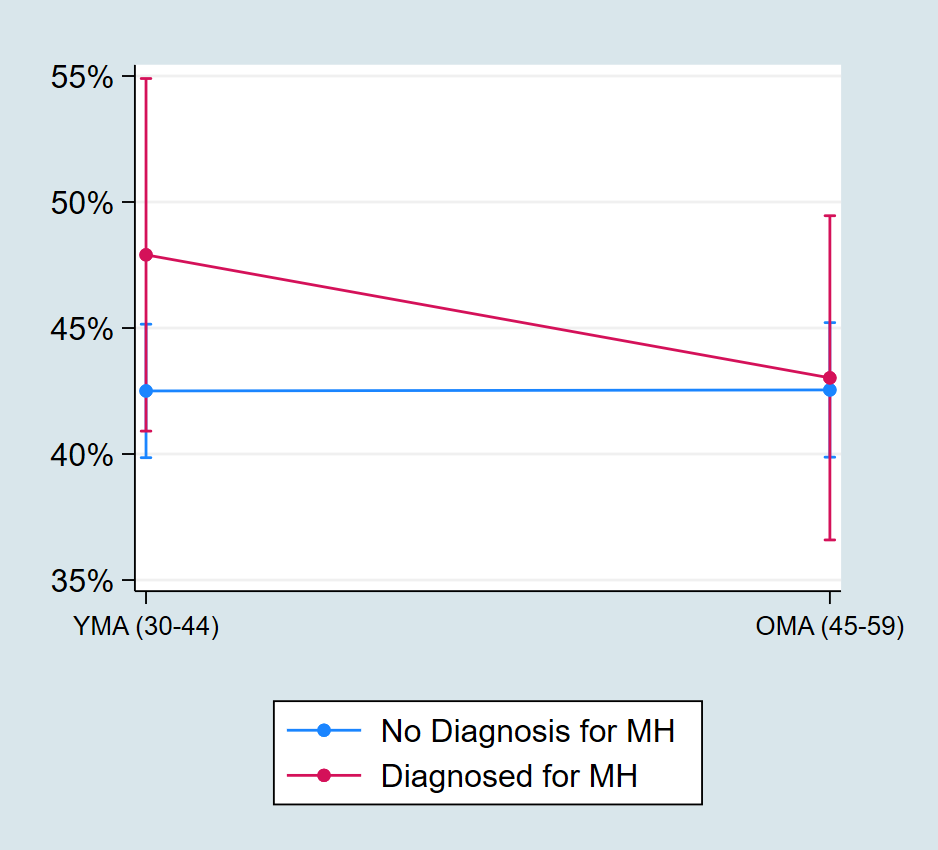


**Supplementary Figure 9.2:** Interaction between Having a Diagnosis and/or Treated for any Mental Health Condition and Age Groups predicting drinking in excess of the Australian National Health and Medical Research Council Guidelines (NHMRC, 2020)

| **Figure 10**: Interactions between Age Groups and Occupational Group (based on ANZSCO) | | | | |
| --- | --- | --- | --- | --- |
| Variables and Categories |  | 95% CI | |  |
|  | **OR** | Lower | Upper | ***p*** |
| Occupational Group (ANZSCO) (RC: Skilled Workers) |  |  |  |  |
| Managers | 1.75 | 1.26 | 2.44 | .001* |
| Professionals | 1.05 | 0.79 | 1.40 | .73 |
| Tech & Trades | 1.83 | 1.35 | 2.48 | <.001** |
| Unskilled Workers | 1.11 | 0.78 | 1.59 | .56 |
| Age Group (RC: YMA) |  |  |  |  |
| OMA | 1.06 | 0.77 | 1.44 | .73 |
| Age Groups x Occupational Group (ANZSCO) (RC: YMA x Skilled Workers) |  |  |  |  |
| OMA x Managers | 0.80 | 0.51 | 1.25 | .33 |
| OMA x Professionals | 0.95 | 0.63 | 1.42 | .80 |
| OMA x Tech & Trades | 0.82 | 0.53 | 1.27 | .37 |
| OMA x Unskilled Workers | 1.07 | 0.64 | 1.80 | .79 |


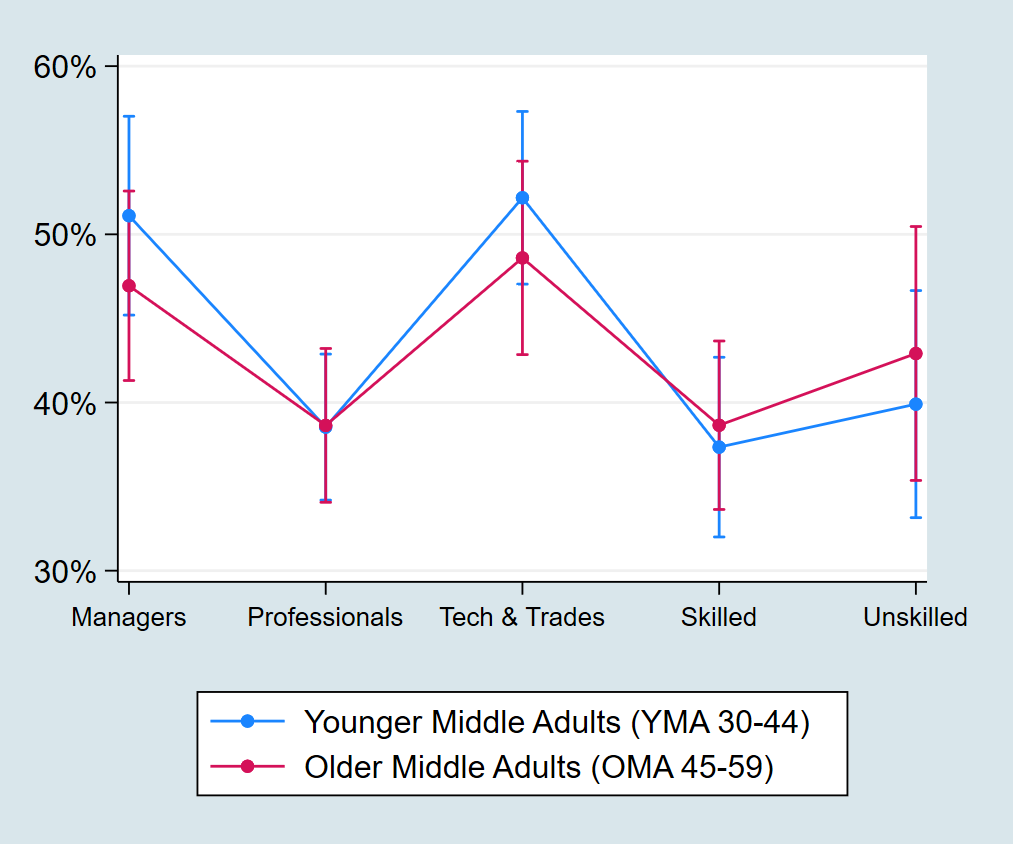


**Supplementary Figure 10.1:** Interaction between Age Groups and Occupational Groups based on the Australian and New Zealand Standard Classification for Occupations (ABS, 2022) predicting drinking in excess of the Australian National Health and Medical Research Council Guidelines (NHMRC, 2020)


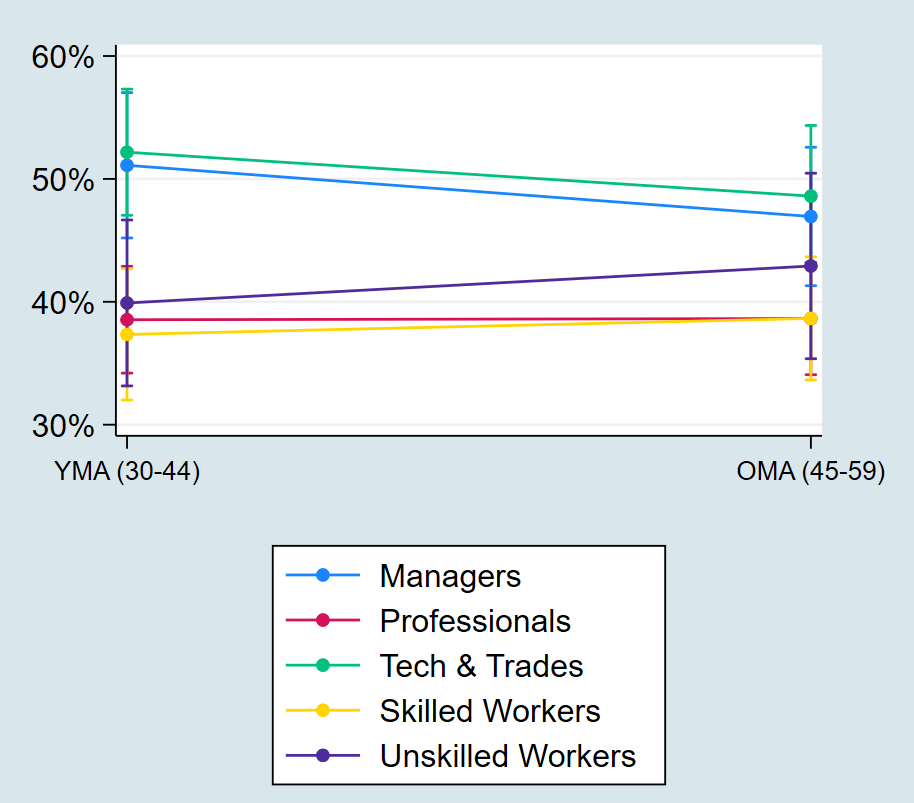


**Supplementary Figure 10.2:** Interaction between Occupational Groups based on the Australian and New Zealand Standard Classification for Occupations (ABS, 2022) and Age Groups predicting drinking in excess of the Australian National Health and Medical Research Council Guidelines (NHMRC, 2020)

| **Figure 11**: Interactions between Age Groups and Household Income | | | | |
| --- | --- | --- | --- | --- |
| Variables and Categories |  | 95% CI | |  |
|  | **OR** | Lower | Upper | ***p*** |
| Household Income (RC: Mid ($1000-1999 per week) |  |  |  |  |
| Don’t Know/Prefer not to say | 0.56 | 0.40 | 0.78 | .001* |
| Low ($999 or less per week) | 0.72 | 0.45 | 1.13 | .15 |
| High ($2000 or more per week) | 1.47 | 1.16 | 1.86 | .001* |
| Age Group (RC: YMA) |  |  |  |  |
| OMA | 1.08 | 0.82 | 1.42 | .59 |
| Age Groups x Household Income (RC: YMA x Mid) |  |  |  |  |
| OMA x Don’t Know/Prefer not to say | 0.93 | 0.58 | 1.50 | .77 |
| OMA x Low ($999 or less per week) | 0.98 | 0.55 | 1.77 | .96 |
| OMA x High ($2000 or more per week) | 0.88 | 0.62 | 1.23 | .45 |


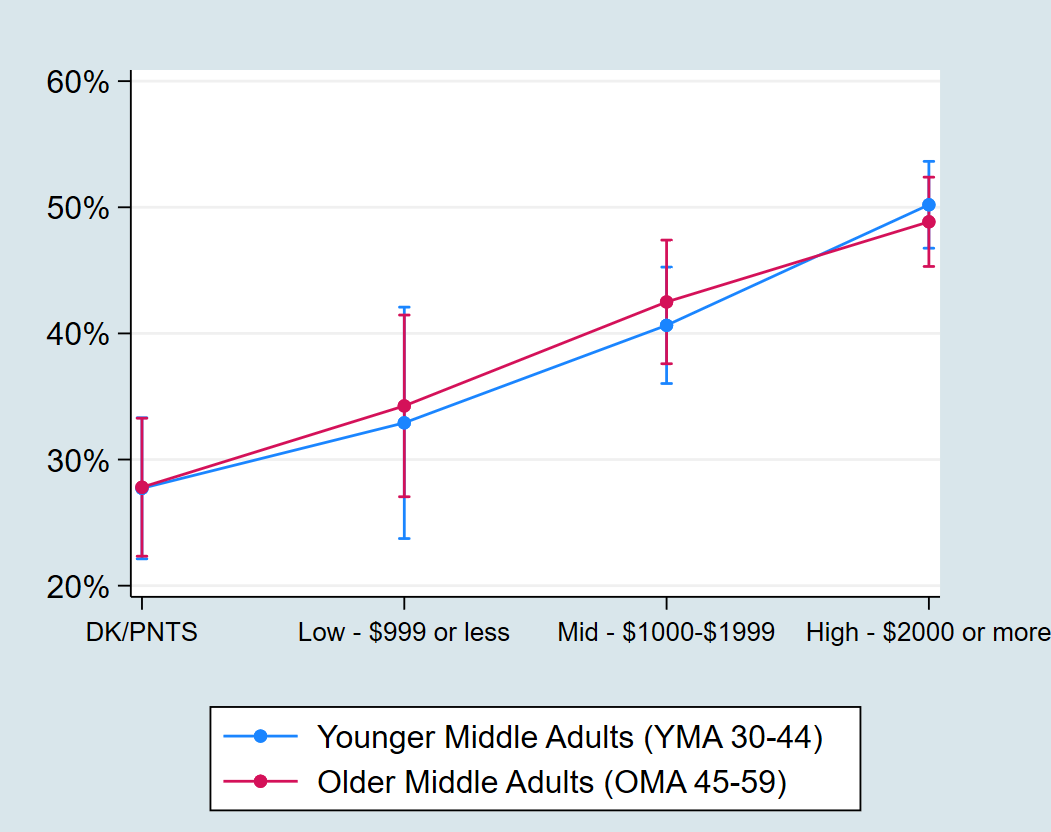


**Supplementary Figure 11.1:** Interaction between Age Groups and Overall Household Income predicting drinking in excess of the Australian National Health and Medical Research Council Guidelines (NHMRC, 2020)

**Supplementary Figure 11.2:** Interaction between Overall Household Income and Age Groups predicting drinking in excess of the Australian National Health and Medical Research Council Guidelines (NHMRC, 2020)


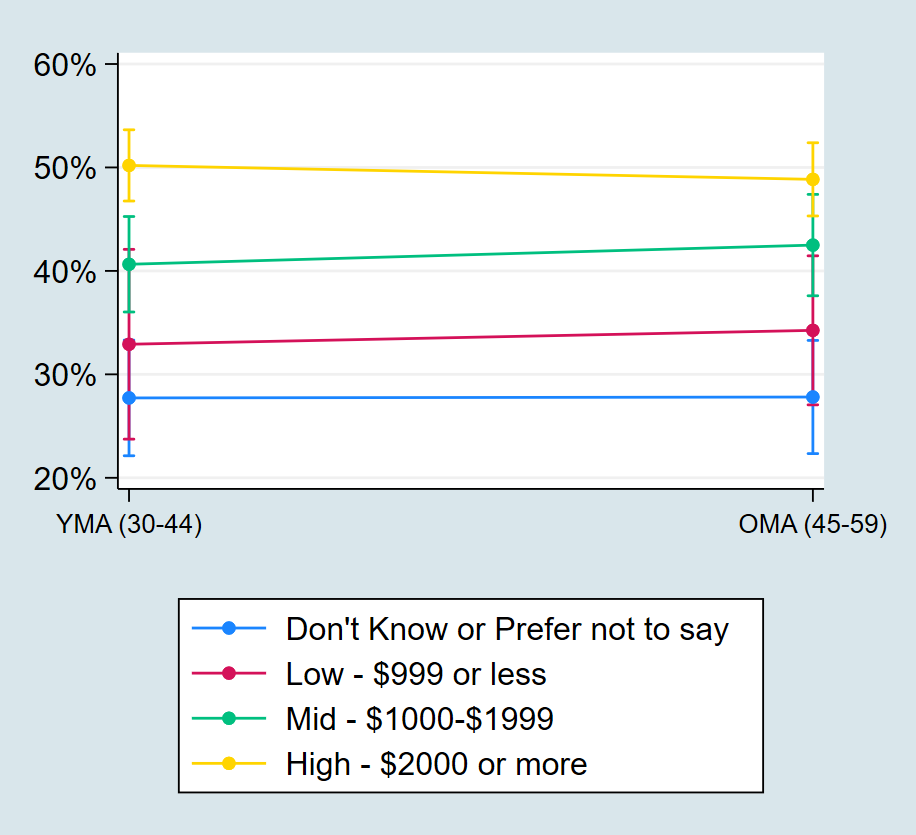

Supplement: Supplementary file 2 — Data S1: Summary of respondents excluded due to incomplete/uninterpretable/invalid responses (values based on raw n). [file DAR-45-0-s001.docx]
